# Wonders of Harbor and Grey Seal Whiskers: Morphology, Natural Frequencies, and 3D Modeling

**DOI:** 10.1002/advs.202500724

**Published:** 2025-04-30

**Authors:** Xingwen Zheng, Amar M. Kamat, Ming Cao, Michael S. Triantafyllou, Ajay Giri Prakash Kottapalli

**Affiliations:** ^1^ Engineering and Technology Institute Groningen Faculty of Science and Engineering University of Groningen Groningen 9747AG Netherlands; ^2^ Institute of Cyber‐Systems and Control Department of Control Science and Engineering Zhejiang University Hangzhou 310027 China; ^3^ Department of Mechanical Engineering Massachusetts Institute of Technology (MIT) Cambridge MA 02139 USA; ^4^ MIT Sea Grant College Program Massachusetts Institute of Technology 77 Massachusetts Avenue Cambridge MA 02139 USA

**Keywords:** 3D modeling, grey seals, harbor seals, natural frequencies, whisker morphologies

## Abstract

Seals can track fish using highly sensitive whiskers; however, the extent to which their morphologically diverse whiskers respond to hydrodynamic signals across frequencies remains unexplored. To address this, the lengths, thicknesses, curvatures, and natural frequencies of whisker arrays in grey seals (*Halichoerus grypus*) and harbor seals (*Phoca vitulina*) are measured. These values are mapped to their corresponding locations on the seal muzzle, and spatial trends (rostral‐caudal and ventral‐dorsal) are analyzed. These findings show that over 50% of whiskers exhibit underwater natural frequencies exceeding 80 Hz, which overlap with hydrodynamic fish trail frequencies (>100 Hz), demonstrating the adaptation of seal whiskers to hydrodynamic signals. Additionally, an open‐access database of 141 full‐length 3D whisker models is established. A streamlined method based on Euler spirals is proposed to fit and map seal whiskers simultaneously. This method evaluates the curvature of the full‐length seal whisker and calculates morphological parameters (e.g., whisker axis and cross‐sectional orientation angles) that are required for 3D whisker construction. The database of 3D seal whiskers offers a valuable resource for researchers in computational fluid dynamics, experimental biology, and sensor technology, supporting multidisciplinary studies of seal whiskers.

## Graphical abstract

Seals detect prey by sensing vortices using whiskers. How whisker morphologies and locations respond to hydrodynamic signals across various frequencies remains unclear. This study measures whiskers’ natural frequencies and morphologies and maps them to whisker locations on the muzzle, providing insights into how seals interpret hydrodynamic signals. Additionally, 144 3D whisker models are created, aiding multidisciplinary studies on seal whiskers.

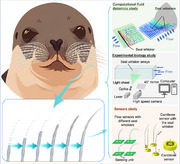

## 1 Introduction

**Figure 1:**
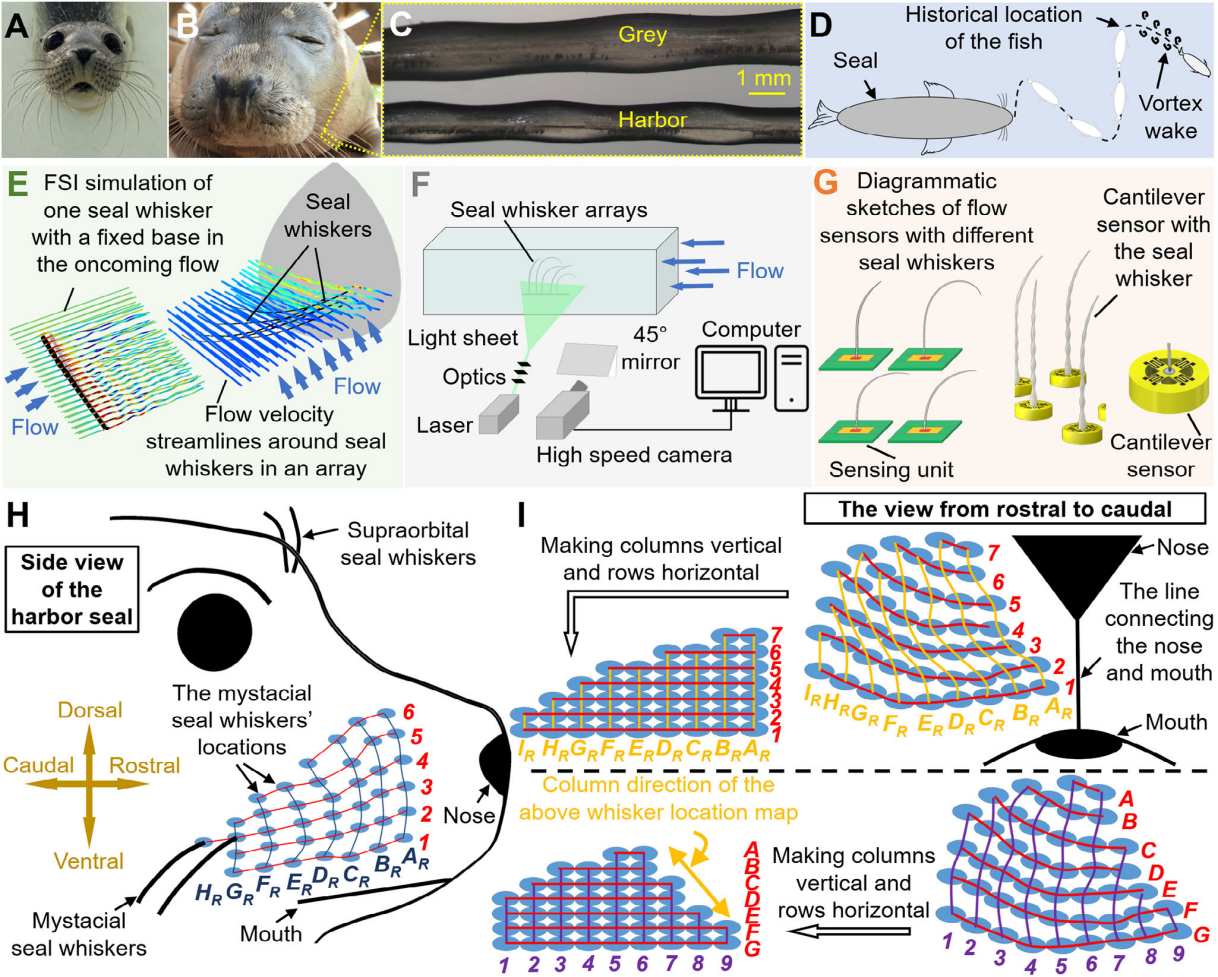
Seal whiskers and their locations on the seal muzzle. A) Harbor seal (*Phoca vitulina*). B) Grey seal (*Halichoerus grypus*). Seal images were provided by Zeehondencentrum, Pieterburen, and the Netherlands and reproduced with permission. C) Close‐up view of seal whiskers showing their unique undulating morphology. Adapted from.^[^ ^18^, ^20^ ^]^ Copyright 2022 Wiley. D) Diagrammatic sketch showing the fish trail‐tracking capability of the seal whisker. E–G) Research topics involving understanding seal‐whisker behavior and functionality from a multidisciplinary perspective, including E) computational fluid dynamics study–exploring flow variations surrounding whiskers arrays to explain the flow sensing mechanism of seal whiskers (adapted from.^[^ ^18^ ^]^ Copyright 2023 Wiley.),^[^ ^10^, ^17^, ^18^ ^]^ F) experimental biology–exploring VIV responses of seal whiskers with different morphologies^[^ ^19^, ^20^, ^21^ ^]^ and G) sensors–referring to measured natural frequencies to select whiskers with specific sensory frequency ranges to design sensors^[^ ^13^, ^20^, ^21^, ^22^, ^23^, ^24^, ^25^, ^26^, ^27^ ^]^ H) Defining the location map of seal whiskers on the seal muzzle using Rows *1*, *2*, *3*, … (from ventral to dorsal) and Columns *A_R_*_,_ *B_R_*_,_ *C_R_*, …. (from rostral to caudal). I) Two examples of the whisker location maps.

After millions of years of natural selection and evolution, many marine organisms have improved their ability to thrive in hostile underwater environments characterized by complex hydrodynamics, limited light, and high turbidity. Leveraging their sensory systems, these animals perceive hydrodynamic stimuli generated by obstacles, prey, and predators, thereby creating situational awareness of the surrounding environment.^[^ ^1^ ^]^ Examples of these sensory systems include the lateral line of fish,^[^ ^2^ ^]^ antennae of shrimp,^[^ ^3^ ^]^ whiskers of pinnipeds (such as harbor seals,^[^ ^4^ ^]^ California sea lions,^[^ ^5^ ^]^ and northern elephant seals^[^ ^6^ ^]^), and arms of octopuses.^[^ ^7^ ^]^ Hyvärinen found that the Saimaa ringed seal (*Phoca hispida saimensis*), which dwells in waters with limited visibility of up to 2 m, possesses extremely well‐developed whiskers.^[^ ^8^ ^]^ The innervation of each whisker is at least ten times greater than that typically found in other mammals,^[^ ^9^ ^]^ resulting in superb sensitivity to underwater disturbances. In addition to the aforementioned well‐developed nerve distributions, some phocid seal species, such as grey seals (*Halichoerus grypus*, **Figure** **1**A) and harbor seals (*Phoca vitulina*, Figure 1B), possess whiskers with distinctive undulating surface morphology (Figure 1C), which resembles beads on a string.^[^ ^10^ ^]^ Moreover, the whiskers exhibit curved structures and variations in thickness from the whisker base to the tip. These whiskers have been shown to contribute significantly to the ultrasensitive underwater sensing ability of these species.^[^ ^11^, ^12^ ^]^

Flow separation occurs when a bluff body, such as a whisker, encounters an oncoming flow or is towed in still water. When the aspect ratio of the body is high, alternating vortices are formed from the boundary layer of the bluff body, creating a downstream Kármán vortex street. The shedding vortices generate periodic alternating inline and transverse loads on the upstream bluff body. When the body is flexible or flexibly mounted, vibrations can be induced in the inline and crossflow directions, particularly if the structural damping is sufficiently low,^[^ ^13^ ^]^ a phenomenon termed vortex‐induced vibration (VIV). A comparative analysis of the VIVs experienced by harbor seal whiskers (with undulations) and California sea lion whiskers (without undulations), measured at the base of the whiskers using piezoelectric sensors, revealed that harbor seal whiskers vibrate six times less than sea lion whiskers.^[^ ^10^ ^]^ The experiments were conducted under identical flow conditions (the same Reynolds number, *Re*). The results highlighted the role of distinctive whisker undulations in suppressing VIVs, as these undulations were the key differentiating factor.

Owing to the VIV suppression capability of seal whiskers, the forward swimming motion of seals does not result in significant whisker vibrations. This, in turn, enhances their sensitivity to hydrodynamic stimuli generated by passing prey. Consequently, undulating seal whiskers can be used to track biologically relevant flows. Such ultrasensitive flow‐stimulus sensing capability was demonstrated by Dehnhardt et al. in behavioral experiments^[^ ^11^, ^12^ ^]^ on a harbor seal (*Phoca vitulina*), which showed that seals could precisely track the hydrodynamic trajectory of a fish swimming approximately 180 m away (Figure 1D). The aforementioned experiments have contributed significantly by inspiring researchers in fields such as robotics, fluid mechanics, and biology, to explore the unique properties of wavy seal whiskers.^[^ ^4^ ^]^ Adachi et al.^[^ ^6^ ^]^ uncovered the decades‐long mystery of how seals locate prey in the deep sea. They demonstrated that northern elephant seals can use their whiskers, with rhythmic movements, to explore their surroundings. They depend on prolonged whisker extensions to detect and capture prey. In addition, bioluminescence accounted for only 20% of the seals’ foraging success, proving that whiskers play a dominant role in prey detection. However, little attention has been given to the mechanism by which seal whiskers respond to hydrodynamic signals across various frequencies encoded in wake trails when tracking prey. Based on concepts from rat whisker literature,^[^ ^14^, ^15^ ^]^ seal whiskers can operate like vibrating cantilevers while engaging in active trail tracking. The frequency modulation of seal whiskers is driven by the hydrodynamic information encoded within tracked trails. Additionally, individual seal whiskers can amplify hydrodynamic signals near their natural frequencies via resonance, analogous to the “differential resonance” theory proposed to explain rats’ behavior when using whisker arrays to encode tactile stimuli.^[^ ^14^, ^15^ ^]^ Unlike terrestrial vibrissae, which primarily respond to low‐frequency mechanical stimuli,^[^ ^16^ ^]^ seal whiskers are likely tuned to higher‐frequency hydrodynamic signals generated by fish wakes. This distinction stems from their adaptation to aquatic environments, where added mass effects reduce resonant frequencies. However, the validity of the “differential resonance” theory for seal whiskers cannot be tested without extensive measurements of the natural frequencies of seal whiskers with diverse morphologies (length, curvature, thickness, etc.) on the seal muzzle and comprehensive comparisons between the measured natural frequencies and the frequencies of the hydrodynamic signals encoded in fish trails. This is the first limitation addressed in this study.

Seal whiskers have attracted considerable attention across various scientific disciplines in recent years owing to their remarkable sensitivity to hydrodynamic stimuli.^[^ ^4^ ^]^ Previous studies have focused on two primary aspects—that is, 1) to understand the role of undulating whisker structures in flow‐sensing mechanisms using computational fluid mechanics (Figure 1E)^[^ ^10^, ^17^, ^18^ ^]^ and experimental biology (Figure 1F)^[^ ^19^, ^21^ ^]^ and 2) the biomimetic potential of seal whiskers in developing whisker‐inspired sensors (Figure 1G)^[^ ^13^, ^20^, ^21^, ^22^, ^23^, ^24^, ^25^, ^26^, ^27^ ^]^ that leverage their undulating surface structures to attain high signal‐to‐noise ratios. Accordingly, the seal whisker community aims to apply whisker‐inspired flow sensors to underwater robots, emulating the trail‐tracking behavior observed in seals^[^ ^11^ ^]^ and enhancing maneuverability and underwater detection through whisker‐aided technology.^[^ ^12^ ^]^

However, a major constraint impeding progress within the research community is the limited access to actual seal whiskers and accurate models of their structures. To address this, Hanke et al.^[^ ^10^ ^]^ proposed a geometric model to construct a 3D undulating structure of seal whiskers. This model has been widely used in diverse studies on seal whiskers, making significant contributions to the field. However, although this model can capture the undulating surface structure of seal whiskers, it does not account for the curvature and taper characteristics evident in actual seal whiskers. Although isolated seal whiskers have been investigated in terms of their morphology, undulations, and the role of undulations in VIV suppression, seal‐whisker studies should ideally examine whisker arrays on the seal muzzle (not only individual whiskers), to comprehensively understand how seals use their whiskers for wake tracking and to realize the potential application of whisker sensor arrays in underwater robots. To address this, it is essential to obtain 3D models of all seal whiskers on the seal muzzle. In recent years, scanning technologies, such as laser and computed tomography scanning, have been employed to capture point‐cloud data representing the geometry of complete seal whiskers. These methods have enabled the creation of seal whisker models with undulations, curvatures, and tapers. However, to obtain 3D models of all seal whiskers on the muzzle, such methods can be expensive. Consequently, the limited access to 3D full‐length whiskers with diverse undulation, curvature, and taper characteristics remains a crucial bottleneck for further studies (the second limitation addressed in this study).

This study contributes to the literature in two ways. First, the natural frequencies and seal‐whisker morphology were analyzed, including their length (measured using image identification technology), thickness (measured using a digital caliper), and curvature (calculated using Euler‐spiral formulations^[^ ^28^ ^]^) for seal whiskers on the seal muzzle across two seal species. The values of natural frequencies and seal whisker morphology were mapped to their corresponding locations on the seal muzzle, and spatial trends (rostral‐to‐caudal and ventral‐to‐dorsal) were analyzed. This study established a comprehensive database of natural frequency and whisker morphology maps. The database can help researchers understand how seal whiskers with diverse morphologies interpret hydrodynamic signals across diverse frequencies while tracking fish trails. Second, we established a database with 141 3D whisker models for two seal species. We constructed the 3D seal whiskers using Euler‐spiral‐fitted whisker axes, Euler‐spiral‐calculated orientation angles of whisker cross‐sections along the whisker axes, and major and minor axes of cross‐sections (calculated by equations that we had proposed in an earlier study^[^ ^18^ ^]^). Researchers in different research fields can access the database, thus advancing their understanding of seal whisker form and function. In the above two critical areas, to evaluate the curvature of the full‐length seal whisker and calculate the morphological parameters (such as the whisker axis and orientation angles of cross‐sections) used to construct 3D seal whiskers, we proposed a streamlined method based on Euler spirals that enables simultaneous fitting and mapping of seal whiskers.

## 2 Results and Discussion

### 2.1 Location Map Defined for Seal Whiskers

For both harbor seals (*Phoca vitulina*) and grey seals (*Halichoerus grypus*), the whiskers are primarily located on the supraorbital (above the eyes) and mystacial (where a mustache might be) regions.^[^ ^29^ ^]^ As few supraorbital whiskers are available, we collected and analyzed mystacial whiskers from the muzzles of deceased seals (*Part A* of the Supporting Text, Supporting Information). Most of the collected mystacial seal whiskers were situated above the seal mouth and beside the seal nose, as viewed from the front facing the head of the seal (Figure 1A,B). In this study, we defined (*Part A* of the Supporting Text, Supporting Information) a whisker location map that reflected their actual distribution on the seal muzzle (Figure 1H).

However, different definitions can result in different whisker location maps. Figure 1I shows two examples of whisker location maps: one (above) defined using our method (*Part A* of the Supporting Text, Supporting Information) and the other (bottom) with different forms of rows and columns that follow the whisker nomenclature established by Dehnhardt and Kaminski^[^ ^30^ ^]^ for whiskers on the seal muzzle. The two whisker location maps are identical in their row forms but differ in their column forms. In this study, we investigated the distribution law of the measured geometric parameters and natural frequencies of seal whiskers. The location map definition method and nomenclature only influence how the distribution law of the whisker measurements is presented. For example, if the columns of the two whisker location maps in Figure 1H are vertical and their rows are horizontal, the column direction of the whisker location map defined in this study was the direction from the bottom right to the top left of the other whisker location map (Figure 1H).

**Figure 2:**
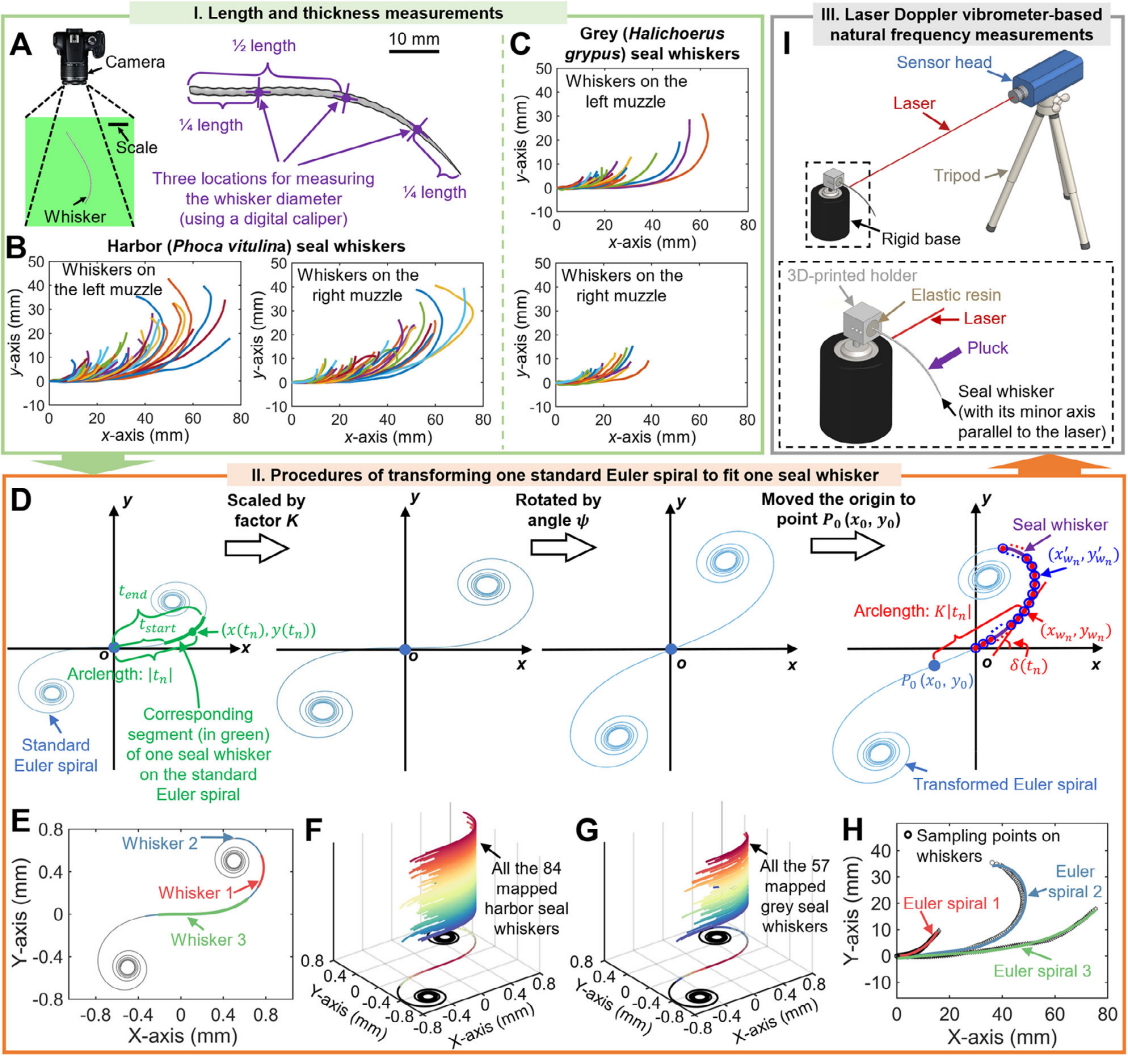
Morphology (length and thickness) and natural‐frequency measurements of seal whiskers and procedures for transforming one standard Euler spiral to fit one seal whisker. A) Diagrammatic sketch showing how to capture the 2D geometry of the seal whisker. The captured image of the seal whisker was then identified as a greyscale image, which was used to collect the coordinates of the whisker shaft sampling points. The seal whisker length was measured based on the collected coordinates. The whisker thickness was evaluated by averaging the whisker diameters measured using a digital caliper at three locations. B,C) Describing the centerlines of seal whiskers by coordinates for harbor and grey seal whiskers. D) The procedures for transforming one standard Euler spiral to fit one seal whisker (illustrated by the purple curve), which is described by the point set $P_{w}=\left\{\left(x_{w_{1}},\;y_{w_{1}}\right),\;\left(x_{w_{2}},\;y_{w_{2}}\right),\;\text{…}\left(x_{w_{n}},\;y_{w_{n}}\right)\right\}$ (marked by red solid points). The points on the transformed Euler used to fit the seal whisker are described by the point set $P_{w}^{\prime }=\;\left\{\left(x_{w_{1}}^{\prime },\;y_{w_{1}}^{\prime }\right),\;\left(x_{w_{2}}^{\prime },\;y_{w_{2}}^{\prime }\right),\;\text{…}\left(x_{w_{n}}^{\prime },\;y_{w_{n}}^{\prime }\right)\right\}$ (marked by blue hollow points). Because *n* = 100, there are 100 red solid points and 100 blue hollow points, but we only draw a few points for illustration. The corresponding segment of the seal whisker on the standard Euler spiral is in green. E) Three seal whiskers mapped on a standard Euler spiral. All F) grey and G) harbor seal whiskers mapped on standard Euler spirals. H) Three seal whiskers (illustrated by 100 sampling points in black) fitted using transformed Euler spirals. I) LDV‐aided natural‐frequency measurements. Adapted from.^[^ ^20^ ^]^ Copyright 2022 Wiley.

**Figure 3:**
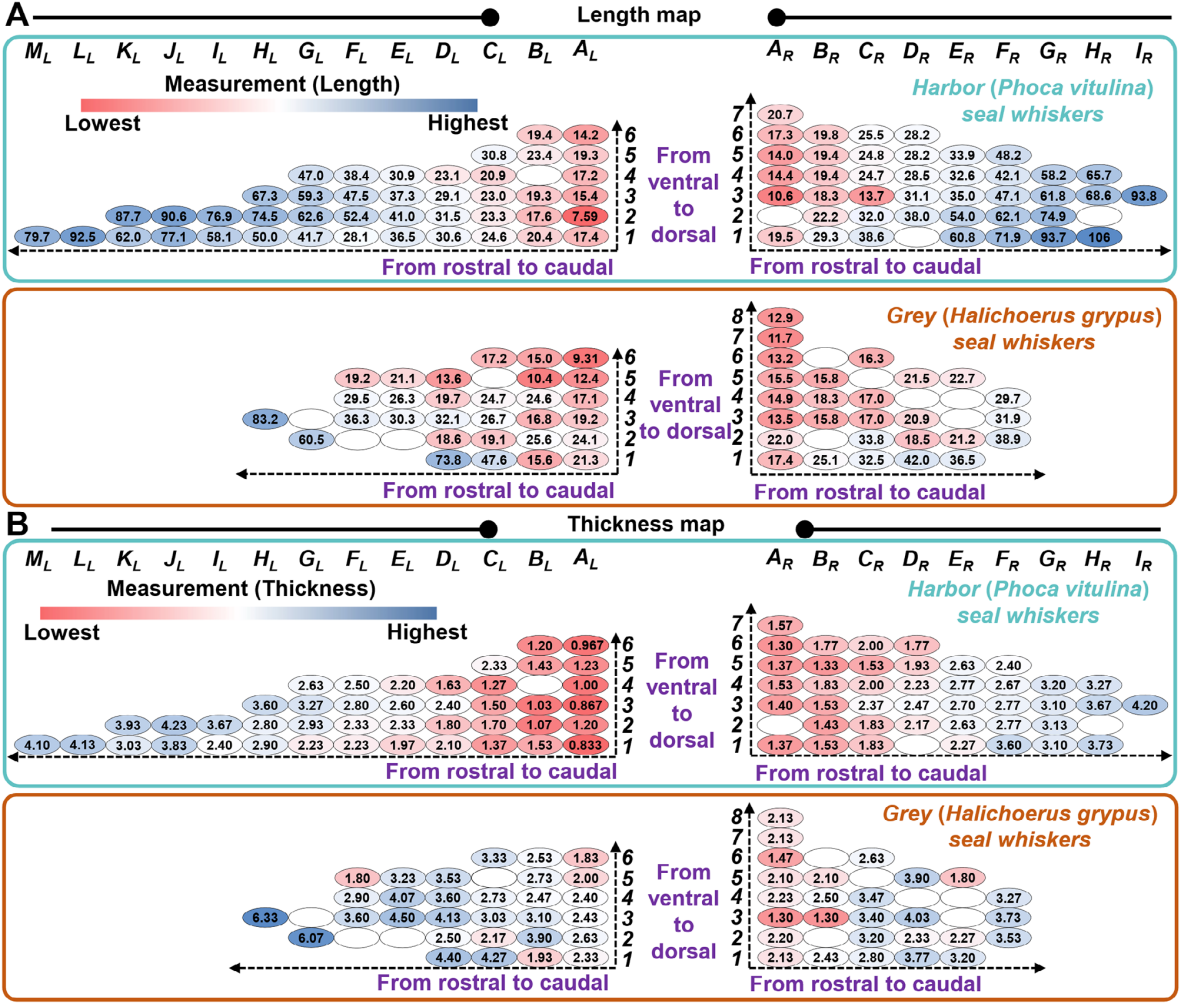
Length and thickness values of harbor and grey seal whiskers. A) Length (mm) values. B) Thickness (×10^−1^ mm) values.

**Figure 4:**
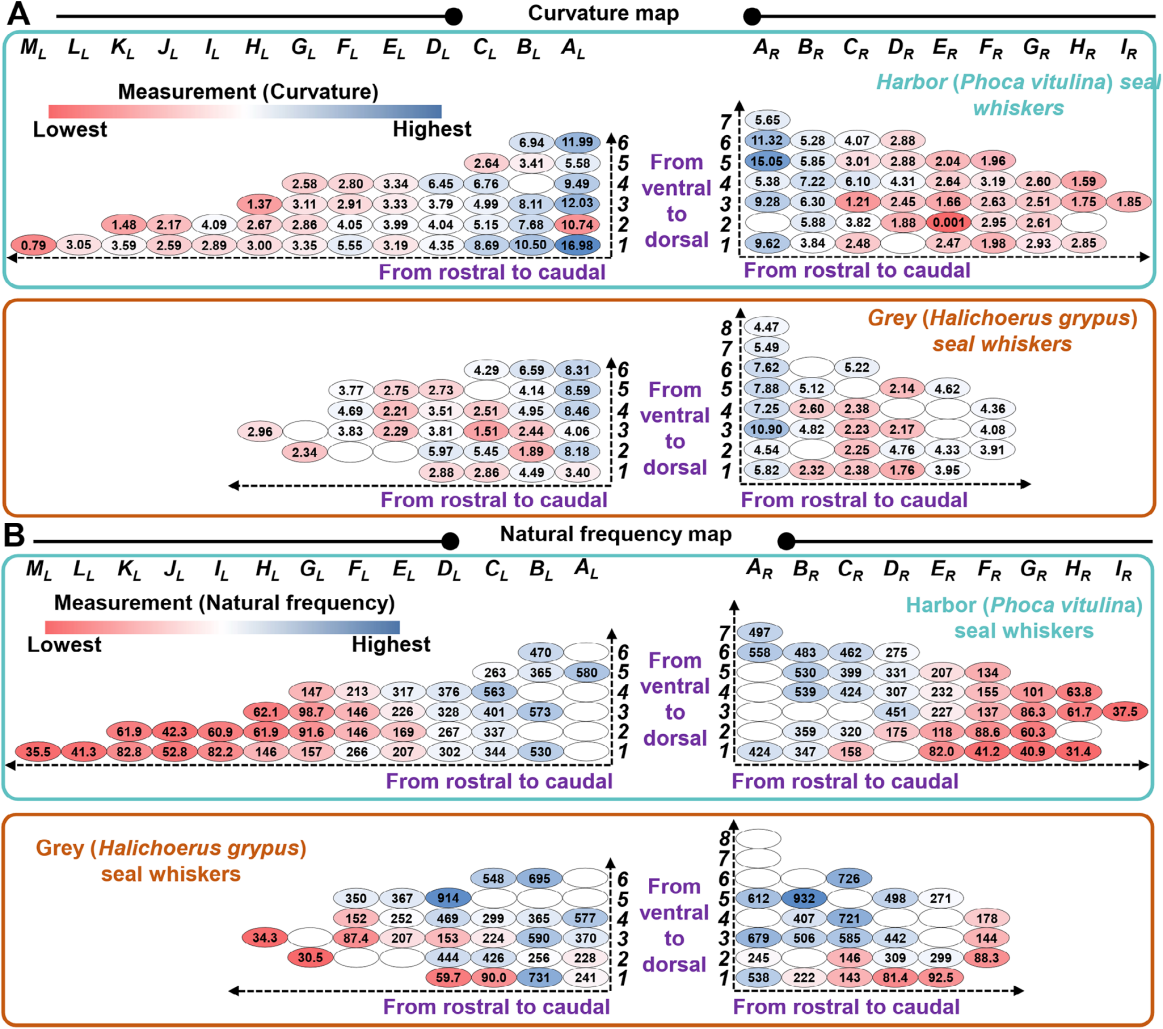
Curvature and natural frequencies of seal whiskers. A) Curvature (×10^−2^ mm^−1^) values of the harbor and grey seal whiskers. B) Natural frequencies (Hz) of the harbor and grey seal whiskers.

**Figure 5:**
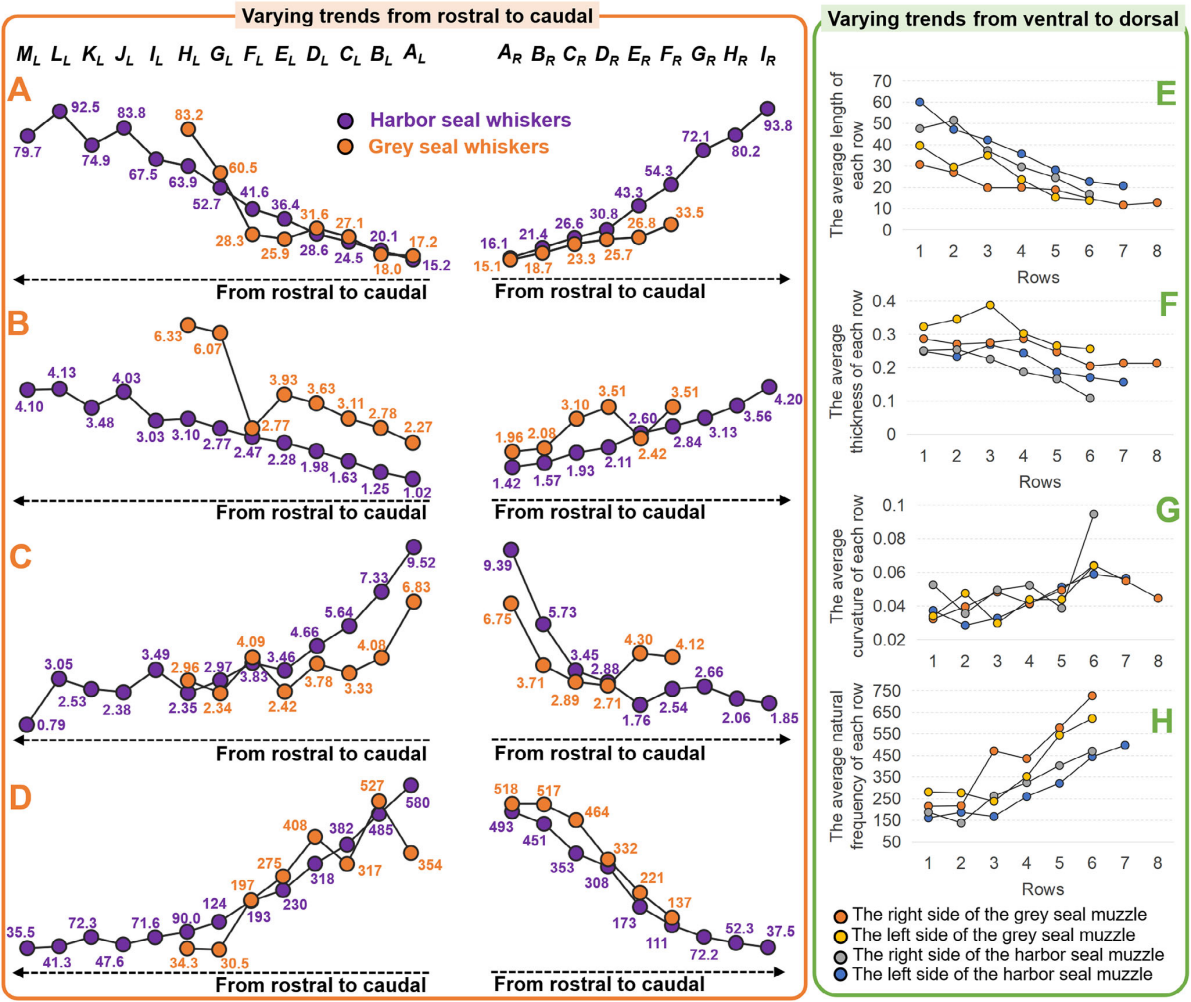
Varying trends of the morphology and natural frequency values of seal whiskers from rostral to caudal and ventral to dorsal on the seal muzzle. A) The average length of each column. B) The average thickness of each column. C) The average curvature of each column. D) The average natural frequency of each column. E) The average length of each row. F) The average thickness of each row. G) The average curvature of each row. H) The average natural frequency of each row.

In this study, we first conducted comprehensive measurements (**Figure** **2**A–I) of the length, thickness, curvature, and natural frequency of the seal whiskers and subsequently located the measured parameters on the defined location map (**Figures** **3**,**4**). Some seal whiskers were not measured since they were naturally lost whiskers (shedding), were found to be broken or cracked (caused while plucking the whisker) at the whisker base, or were already used as elements of whisker‐sensors. However, these not‐measured whiskers would not affect the qualitative varying trends of the measured natural frequency and whisker morphology because only 1–2 whiskers were not measured in each row and each column (Figures 3, 4). The morphologies and natural frequencies were located on identical whisker location maps, ensuring the correspondence between the morphologies and natural frequencies. Moreover, the varying trends (**Figure** **5**A–H) of the morphology and natural frequency values of seal whiskers from rostral to caudal and ventral to dorsal were analyzed.

### 2.2 Seal‐Whisker Length

Details regarding the measurement methods of the whisker length and thickness are provided in *Part A* of the Experimental Section. The lengths of the harbor seal whiskers ranged from 7.59 to 106 mm (Figure 3A), averaging 40.7 mm. The number of seal whiskers on both sides was not exactly the same because this was an actual seal feature. By observing columns from rostral to caudal, the average whisker length increased from 16.1 (Column *A_R_*) to 93.8 mm (Column *I_R_*) on the right muzzle, and from 15.2 (Column *A_L_*) to 92.5 mm (Column *L_L_*) on the left muzzle for harbor seal whiskers (Figures 3A,5A). The length of grey seal whiskers ranged from 9.31 to 83.2 mm with an average of 24.7 mm (Figure 3A). By observing columns from rostral to caudal, the average whisker length increased from 15.1 mm (Column *A_R_*) to 33.5 mm (Column *F_R_*) on the right muzzle (Figures 3A,5A). Moreover, the average whisker length increased from 17.2 mm (Column *A_L_*) to 83.2 mm (Column *H_L_*) on the left muzzle (Figures 3A,5A). These observations indicate rostral‐to‐caudal length increases. Moreover, by observing rows from ventral to dorsal, the average whisker length decreased from 60 (Row 1) to 20.7 mm (Row *7*) on the right muzzle (Figure 5E) and 47.6 (Row *1*) to 16.8 mm (Row *6*) on the left muzzle for harbor seal whiskers (Figure 5E). Besides, the average whisker length decreased from 30.7 (Row *1*) to 12.9 mm (Row *8*) on the right muzzle (Figure 5E), and 39.6 (Row *1*) to 13.8 mm (Row *6*) on the left muzzle for grey seal whiskers (Figure 5E). Seals have different whisker lengths depending on their age. Moreover, seals renew and replace their whiskers annually. Consequently, our results may be biased, as we only presented length measurements for one seal for each species in this study, which is a study limitation.

In this study, the whisker length was calculated using the image recognition technology‐identified sampling points (*Part A* of the Experimental Section). The sampling points were sufficient to completely describe the curved structure of the whiskers (Figure 2B,C), failing which, the sum of the point‐to‐point distances of the identified coordinates of the whisker shaft would differ considerably from the whisker length. Accordingly, we used at least 500 sampling points (corresponding to the shortest whisker) of the whisker shaft for the length calculation, whereas fewer than 100 sampling points (corresponding to the longest whisker) were used in our previous work.^[^ ^31^ ^]^ The greater the number of sampling points used, the more accurate the calculated whisker length. The lengths of harbor seal whiskers located at (Row *3*, Column *B_L_*), (Row *5*, Column *A_L_*), (Row *4*, Column *B_R_*), and (Row *5*, Column *B_R_*) were approximately 19.3 or 19.4 mm (Figure 3A), but they exhibited diverse curvature (corresponding to diverse whisker axis shapes) and thickness values. Moreover, owing to space limitations in Figures 3 and 4, the measurements (length, thickness, curvature, and natural frequency) are presented using scientific notation with limited significant digits. Dataset S1 (Supporting Information) presents other forms (with more significant digits) of the numerical results of measurements in Figures 3 and 4. The raw data used to calculate whisker length are presented in Dataset S2 (Supporting Information).

Graff et al.^[^ ^32^ ^]^ predicted harbor seal whiskers’ length (ranging from approximately 10–120 mm) using one equation proposed based on their measurements of real seal whiskers. They located the predicted length values in a whisker location map defined based on the established whisker nomenclature (Figure 1I) proposed by Dehnhardt and Kaminski^[^ ^30^ ^]^ for harbor seals. The correspondence between the whisker location maps defined in^[^ ^32^ ^]^ and this study is similar to the two examples of whisker location maps in Figure 1I; that is, our column direction is the bottom‐right‐to‐top‐left direction of their whisker location map (Figure 2F in ref. [32]). Both^[^ ^32^ ^]^ and this study demonstrated the length increase from rostral to caudal of the harbor seal whiskers. They also described the whisker length values as a function of whisker position,^[^ ^32^ ^]^ providing a valuable reference to the quantitative relationship between whisker location and morphology for research communities focused on seal whisker research.

### 2.3 Seal‐Whisker Thickness

Seal whiskers have elliptical rather than circular cross‐sections. In our previous work,^[^ ^21^ ^]^ we measured the major and minor axes of their elliptical cross‐sections along the length of two full‐length seal whiskers based on cross‐sectional slices of scanned CAD models. However, measuring the diameter of all cross‐sections along the length of all whiskers was impractical because the CAD models of the 84 harbor seal whiskers and 57 grey seal whiskers used in this study were not scanned. In fact, obtaining CAD models of seal whiskers using blue‐light scanning technology is costly (200 € for scanning one seal whisker).^[^ ^21^ ^]^ Consequently, we addressed the issue of high cost by developing CAD models of 3D seal whiskers in *Part G* of the Results and Discussion section. Instead of measuring the major and minor of all cross‐sections along the whisker length, we measured the whisker minors at three locations along the whisker length (*Part A* of the Experimental Section), and the average of three measurements was calculated as an index to evaluate the size level of the whisker thickness. However, the index (i.e., the whisker thickness analyzed in this study) did not equal the accurate whisker thickness value, because the major and minor axes exhibit 3D undulations along the whisker length.^[^ ^18^, ^21^ ^]^ Nonetheless, it was feasible for comparing the size levels of the thickness of diverse whiskers and then analyzing their varying trends from rostral to caudal and rostral to caudal.

The thickness of harbor seal whiskers ranged from 0.0833 to 0.420 mm (Figure 3B), with an average of 0.228 mm. By observing columns from rostral to caudal, the average thickness increased from 0.142 (Column *A_R_*) to 0.420 mm (Column *I_R_*) on the right muzzle for harbor seal whiskers (Figures 3B,5B). Similarly, it increased from 0.102 (Column *A_L_*) to 0.410 mm (Column *M_L_*) on the left muzzle (Figures 3B,5B). The grey seal‐whisker thickness ranged from 0.130 to 0.633 mm (Figure 3B), with an average of 0.295 mm. By observing columns from rostral to caudal, the average thickness increased from 0.196 (Column *A_R_*) to 0.351 mm (Column *F_R_*) on the right muzzle and from 0.227 (Column *A_L_*) to 0.633 mm (Column *H_L_*) on the left muzzle (Figures 3B,5B). The aforementioned analyses showed that whisker thickness increased from rostral to caudal. Moreover, by analyzing the average thickness of each row, it decreased from 0.249 (Row *1*) to 0.157 mm (Row *7*) on the right muzzle for harbor seal whiskers (Figure 5F). Besides, it increased from 0.251 (Row *1*) to 0.108 mm (Row *6*) on the left muzzle (Figure 5F). Similarly, the grey seal‐whisker thickness decreased from 0.287 (Row *1*) to 0.213 mm (Row *8*) on the right muzzle and from 0.323 (Row *1*) to 0.257 mm (Row *6*) on the left muzzle (Figure 5F). The above varying trends from rostral to caudal and from ventral to dorsal were similar to the varying trends of the whisker length, indicating a high correlation between whisker thickness and whisker length.

### 2.4 Simultaneous Fittings and Mappings of Seal Whiskers Based on Euler Spirals

Towal et al.^[^ ^33^ ^]^ validated that the shaft of a rat whisker could be fitted using a quadratic curve.^[^ ^28^ ^]^ Furthermore, Starostin et al.^[^ ^34^ ^]^ demonstrated that all whiskers on the rat muzzle could be mapped onto a standard Euler spiral. Subsequently, Dougill et al.^[^ ^35^ ^]^ quantified the whisker sizes and shapes of 19 mammalian species—including the house mouse, red fox, and common shrew—describing the curvature of the whiskers using the Euler spiral^[^ ^28^ ^]^ (a curve whose curvature varies linearly with its arc length). Dougill et al.^[^ ^35^ ^]^ and Luo and Hartman^[^ ^36^ ^]^ used an equation with two coefficients (*A* and *B*)—that is, *κ*(*s*) = *As *+ *B*, where *κ* denotes the curvature, *s* denotes the length, and *A* and *B* denote constants—to describe the Euler‐spiral‐fitted seal whisker. Based on this equation, Starostin et al.^[^ ^34^ ^]^ introduced a detailed mathematical model for fitting whiskers of mammalian species using Euler spirals (“fitting procedure”) and mapping whiskers to one standard Euler spiral (“mapping procedure”). Although the fittings and mappings were efficient, they required further mathematical derivations, based on the equation (*κ*(*s*) = *As *+ *B*), in the fitting^[^ ^33^ ^]^ and mapping procedures.^[^ ^34^ ^]^ Moreover, the fitting^[^ ^33^ ^]^ and mapping^[^ ^34^ ^]^ procedures were completed using two separate mathematical modeling steps, which are described in detail in^[^ ^33^ ^]^ and,^[^ ^34^ ^]^ respectively.

Unlike prior methods requiring separate fitting and mapping steps^[^ ^34^, ^35^, ^36^ ^]^ we transformed one standard Euler spiral (Equation (1)) to obtain one transformed Euler spiral (Equation (2)), then proposed a streamlined approach using nonlinear identification of the transformed Euler spiral equation to simultaneously fit and map one seal whisker (Figure 2D) that described by image identification technology‐collected coordinates (*Part B* the of Experimental Section). The transformed Euler spiral (Equation (2)) can be obtained by scaling up or down, rotating, or moving the standard Euler spiral (Equation (1), Figure 2D). The nonlinear fitting of Equation (2) outputs six coefficients (*K*, ψ, *t_start_*, *t_end_*, *x*_0_, *y*_0_), among which *K*, ψ, *x*_0_, and *y*_0_ reflect transformation procedures from one standard Euler spiral (corresponding to the “fitting procedure”), and *t_start_* and *t_end_* are the arc lengths (calculated from the origin of the standard Euler spiral (Figure 2E)) corresponding to the starting and ending points of the seal whisker mapped on a standard Euler spiral (corresponding to the “mapping procedure”). The above fitting and mapping procedures of seal whiskers based on Euler spirals are summarized in a flowchart in Figure S1 (Supporting Information). The average ($\bar{t}=\frac{t_{start}+t_{end}}{2}$) of the *t_start_* and *t_end_* values reflected the location of the center of one seal whisker when it was mapped on the standard Euler spiral. The larger the $\bar{t}$ value, the farther away the location of the center of the mapped whisker is from the origin of the standard Euler spiral (Figure 2E). The difference (Δ*t*   =  *t_end_*  −  *t_start_* ) between the *t_start_* and *t_end_* values reflected the distribution range of one seal whisker when it was mapped on the standard Euler spiral. In other words, the Δ*t* value reflected the arc length of the seal whisker mapped on the standard Euler spiral. In *Part B* of the Supporting Text (Supporting Information), we have located the $\bar{t}$ and Δ*t* values in the defined whisker location map (Figure S2, Supporting Information) and analyzed their varying trends (Figure S3, Supporting Information) from rostral to caudal.

Based on the above descriptions, our method improves efficiency by unifying the fitting and mapping procedures of seal whiskers into one step—that is, the nonlinear fitting of Equation (2). This was an improvement compared to previous studies^[^ ^34^, ^35^, ^36^ ^]^ which completed the fitting and mapping procedures in two separate steps.^[^ ^34^ ^]^ Moreover, compared to^[^ ^34^, ^35^, ^36^ ^]^ which needed the necessary mathematical derivations based on the proposed equation (*κ*(*s*) = *As *+ *B*)—Equation (2) in our method did not require further mathematical derivations when completing the “fitting procedure” and “mapping procedure”. Additionally, the coefficients *K* and ψ could be used to calculate the curvature (based on Equation (3)) of the seal whisker along its arclength and the angle (based on Equation (4)) between the tangent of the seal whisker and the *ox* axis (Figure 2D). The latter can be further used to calculate the orientation angles of whisker cross‐sections along the whisker axes in the construction of 3D seal whiskers, as discussed in *Part G* of the Results and Discussion section.

For each seal whisker, its corresponding transformed Euler spiral and mapped segment onto the standard Euler spirals are presented in Dataset S3 (Supporting Information). The root‐mean‐squared error (*RMSE_w_*, Equation (5), *Part B* of the Experimental Section) was calculated between the point set (collected using image identification technology) of the seal whisker and the point set on the corresponding transformed Euler spiral. The *RMSE_w_* value (Figure S4, Supporting Information) is a common indicator for evaluating the registration accuracy between two point sets. In this study, it was used to evaluate the error in fitting one seal whisker (described by the point set $P_{w}=\left\{\left(x_{w_{1}},\;y_{w_{1}}\right),\;\left(x_{w_{2}},\;y_{w_{2}}\right),\;\text{…}\left(x_{w_{n}},\;y_{w_{n}}\right)\right\}$, Figure 2D) using the transformed Euler spiral (described by the point set $P_{w}^{\prime }=\left\{\left(x_{w_{1}}^{\prime },\;y_{w_{1}}^{\prime }\right),\;\left(x_{w_{2}}^{\prime },\;y_{w_{2}}^{\prime }\right),\;\text{…}\left(x_{w_{n}}^{\prime },\;y_{w_{n}}^{\prime }\right)\right\}$, Figure 2D). The average *RMSE_w_* values (Figure S4, Supporting Information) were 0.757 and 0.536 mm for harbor and grey seal whiskers, respectively. The aforementioned *RMSE_w_* values were only 1.86% and 2.17% of the average length of harbor (40.67 mm) and grey seal whiskers (24.69 mm), respectively, indicating that each seal whisker could be fitted using a transformed Euler spiral with minor errors. Evidently, all harbor or grey seal whiskers could be mapped onto a standard Euler spiral (Figure 2E). The whiskers covered most of the areas of the Euler spiral (Figure 2F,G), which was consistent with the observations in previous studies^[^ ^34^, ^35^, ^36^ ^]^ The extensive coverage (Figure 2F) of all seal whiskers mapped on the standard Euler spiral was indicative of the extensive curvature range of seal whiskers on the seal muzzle, which is further investigated in the following section.

### 2.5 Seal‐Whisker Curvature

One of our motivations for studying Euler spirals was to use the Euler spiral theory as a tool to calculate the curvature along the length of one seal whisker. Dougill et al.^[^ ^35^ ^]^ studied 61 harbor seal whiskers, and Luo and Hartman^[^ ^36^ ^]^ studied 114 harbor seal whiskers using Euler spirals. They used the previously mentioned linear equation (*κ*(*s*) = *As *+ *B*), which uses two coefficients to fit the whisker curvature as a linear function of normalized whisker length. In contrast to these studies,^[^ ^35^, ^36^ ^]^ this study calculated the whisker curvature using Equation (3) (*Part B* of the Experimental Section),^[^ ^28^ ^]^ originating from the fundamental theory of the Euler spiral^[^ ^34^ ^]^ and with only one coefficient (*K*). The value of coefficient (*K*) could be obtained simultaneously during the nonlinear identification of Equation (2). The curvature of the transformed Euler spiral‐fitted seal whisker varies linearly (Equation (3)) with the arc length of the whisker.^[^ ^28^ ^]^ Consequently, the average value of the curvatures at the start and end points of the transformed Euler‐spiral‐fitted seal whisker was calculated (by Equation (6)) as an index for evaluating the curvature size (Figure 4A) of the full‐length whisker. Another motivation for studying Euler spirals was that the Euler spiral theory enables the calculation of the orientation angles of the cross‐sections of one seal whisker along its length. The calculated orientation angles were used to construct a 3D seal whisker. More details are presented in *Part G* of the Results and Discussion section.

For the harbor seal whiskers’ curvatures—which ranged from 0.00001 to 0.1698 mm^−1^, with an average curvature of 0.0447 mm^−1^ (Figure 4A)—the average curvature along the columns from rostral to caudal decreased from 0.0939 (Column *A_R_*) to 0.0185 mm^−1^ (Column *I_R_*) on the right muzzle and from 0.0952 (Column *A_L_*) to 0.0079 mm^−1^ (Column *M_L_*) on the left muzzle (Figures 4A,5C). The harbor seal whisker (Column *E_R_*, Row *2*, Figure 4A) with a curvature of 0.00001 mm^−1^ was a rare case. Its tip was curled opposite the curved direction of the whisker axis (Dataset S3, Supporting Information). It was fitted using an almost straight line and mapped to a segment close to the origin of one standard Euler spiral (Dataset S3, Supporting Information), resulting in an error in fitting when using a transformed Euler spiral. If this seal whisker is not considered, the curvatures of the harbor seal whiskers range from 0.0079 to 0.1698 mm^−1^, with an average curvature of 0.0452 mm^−1^. For the grey seal whiskers’ curvatures—which ranged from 0.0151 to 0.1090 mm^−1^, with an average curvature of 0.0430 mm^−1^—the average curvature along the columns decreased slightly from 0.0683 (Column *A_L_*) to 0.0296 mm^−1^ (Column *H_L_*) on the left muzzle (Figures 4A,5C). Moreover, the average curvature decreased from 0.0675 (Column *A_R_*) to 0.0271 mm^−1^ (Column *D_R_*) before increasing to 0.0412 mm^−1^ (Column *F_R_*), from rostral to caudal on the right muzzle (Figures 4A,5C). Although this was not a continuously decreasing trend, considering the decreasing trends (from rostral to caudal) of the whisker curvature of all harbor seal whiskers and left‐muzzle grey seal whiskers, we concluded that the closer the whisker was to the rostral, the larger was its curvature.

Moreover, for the average whisker curvatures along the rows from ventral to dorsal, the varying trend was insignificant for both harbor and grey seal whiskers. However, there was still a slight increase in the average curvatures. For the harbor seal whiskers’ curvatures, their average curvature along the rows from ventral to dorsal increased to 0.0589 mm^−1^ (Row *6*) on the right muzzle and to 0.0946 mm^−1^ (Row *6*) on the left muzzle (Figure 5G). For the grey seal whiskers’ curvatures, their average curvature along the rows increased to 0.0642 mm^−1^ (Row *6*) on the right muzzle and to 0.0640 mm^−1^ (Row *6*) on the left muzzle (Figure 5G).

### 2.6 Natural Frequencies of Seal Whiskers

Overall, the average natural frequencies of the harbor and grey seal whiskers were 240.47 and 360.67 Hz, respectively. For harbor seal whiskers, by observing the natural frequency values (Figure 4B) at columns from rostral to caudal, the average natural frequency (the measurement method of which is in *Part C* of the Experimental Section) decreased from 492.69 (Column *A_R_*) to 37.53 Hz (Column *I_R_*) along the columns on the right muzzle and from 580.48 (Column *A_L_*) to 35.45 Hz (Column *M_L_*) on the left muzzle (Figures 4B,5D). For grey seal whiskers, by observing the natural frequency values (Figure 4B) at columns from rostral to caudal, the average natural frequency decreased from 518.47 (Column *A_R_*) to 136.86 Hz (Column *F_R_*) on the right muzzle and from 354.12 (Column *A_L_*) to 34.25 Hz (Column *H_L_*) on the left muzzle (Figures 4B,5D).

By observing the natural frequency values at rows from ventral to dorsal, for harbor seal whiskers, the average natural frequency increased from 160.53 (Row *1*) to 496.95 Hz (Row *7*) along the rows on the right muzzle and from 187.23 (Row *1*) to 469.62 Hz (Row *6*) on the left muzzle (Figure 5H). For grey seal whiskers, the average natural frequency increased from 215.28 (Row *1*) to 726.05 Hz (Row *6*) on the right muzzle and from 280.56 (Row *1*) to 621.23 Hz (Row *6*) on the left muzzle (Figure 5H). The opposite varying trends of whisker length and natural frequency along columns and rows indicate that whiskers with larger lengths may sense hydrodynamic stimuli with frequencies close to the larger natural frequencies. Assuming the “differential resonance” theory^[^ ^14^, ^15^ ^]^ to be valid, seal whiskers with higher natural frequencies have evolved to be more attuned to the higher frequencies typically emitted by smaller fish.

Shatz and de Groot^[^ ^37^ ^]^ conducted natural‐frequency measurements on harp‐seal whiskers of various lengths (33–105 mm) using a variable‐frequency acoustic source to stimulate them in an airborne environment. The recorded natural frequencies were in the range of 20–200 Hz. However, only a limited number of seal whiskers (≈13) were employed in these measurements.^[^ ^37^ ^]^ In this study, we conducted extensive measurements on the natural‐frequency distribution of seal whiskers across two distinct seal species—that is, 84 whiskers for the harbor seal and 57 whiskers for the grey seal. The measured natural frequencies ranged from 35.45 to 580.5 Hz for harbor seal whiskers (Figure 4B) and 30.45 to 931.9 Hz for grey seal whiskers (Figure 4B), covering the 30–175 Hz frequency range measured on isolated harbor seal whiskers in air using a mini‐shaker apparatus.^[^ ^38^ ^]^

**Figure 6:**
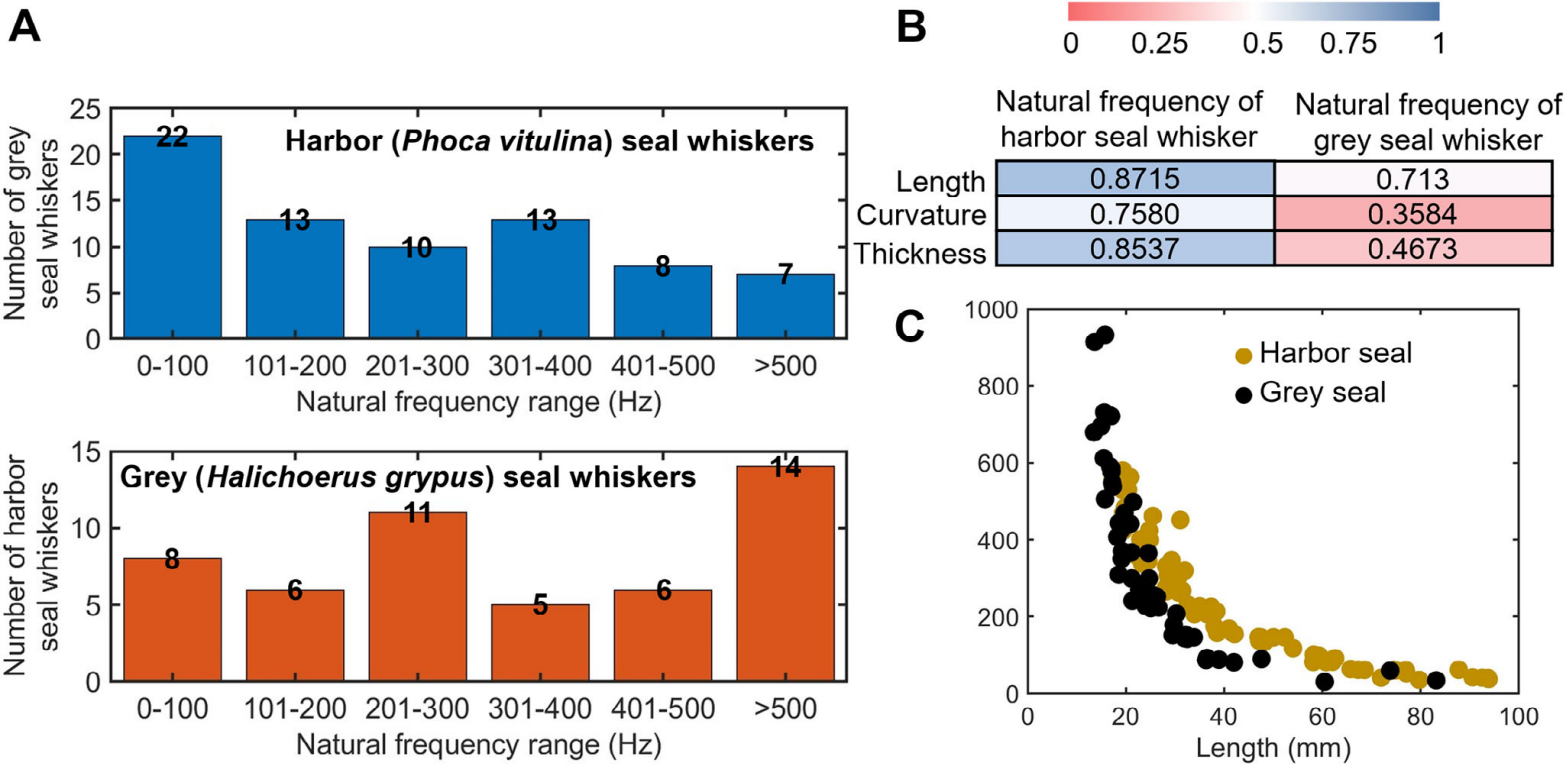
Additional analyses of the natural frequencies of seal whiskers. A) Number of the harbor and grey seal whiskers in various natural frequency ranges. B) Correlation between natural frequency and morphology (length, curvature, and thickness). C) Natural frequency–length relationship for harbor and grey seal whiskers.

However, the above natural frequencies were measured in air. The natural frequencies of seal whiskers decrease underwater primarily due to the added mass effect,^[^ ^20^ ^]^ while hydrodynamic damping and fluid‐structure interactions (e.g., viscosity‐induced energy dissipation) further suppress vibrational amplitudes. These effects collectively determine the underwater vibration response of whiskers. Our previous work^[^ ^20^ ^]^ demonstrated that harbor and grey seal whiskers exhibit 40.86% and 48.15% reductions in natural frequencies underwater, respectively, compared to their in‐air measurements. Following this attenuation pattern, an in‐air frequency of 200 Hz translates to underwater frequencies of 81.72 Hz for harbor seal whiskers and 96.3 Hz for grey seal whiskers. As shown in **Figure** **6**A, 52% of harbor seal whiskers and 72% of grey seal whiskers displayed in‐air natural frequencies exceeding 200 Hz. This implies that 52% and 72% of these whiskers maintain underwater natural frequencies surpassing 81.72 and 96.3 Hz, respectively. Consequently, over 50% of whiskers in both species retain underwater natural frequencies above 80 Hz. This range overlaps the 100–300 Hz frequencies measured in supraorbital whiskers of harbor seals during fish trail tracking^[^ ^39^ ^]^ and hydrodynamic fish trail frequencies (>100 Hz).^[^ ^40^ ^]^ This adaptability could help seals track fish under the water.^[^ ^11^, ^12^ ^]^

Correlation analyses (Figure 6B) encompassing the measured length, curvature, thickness, and natural frequency showed that the natural frequency exhibited a strong correlation with the whisker length for both seal species. Further analyses (Figure 6C) revealed that the natural frequency of the seal whisker decreased with whisker length. Assuming the “differential resonance”^[^ ^14^, ^15^ ^]^ theory to be valid, the natural frequency of a seal whisker could be excited by specific underwater disturbances, such as a fish wake containing unsteady features with frequencies within the natural frequency range of the seal whisker. Using the results shown in Figure 6C, we can infer that long seal whiskers with low natural frequencies may primarily respond to—that is, be excited by—low‐frequency underwater disturbances. By contrast, short seal whiskers with high natural frequencies may primarily respond to—that is, be excited by—underwater disturbances of high frequency. However, further investigations should be conducted to prove this inference, as the vibration frequencies of seal whiskers in underwater disturbances are not only affected by the “differential resonance”^[^ ^14^, ^15^ ^]^ between the natural frequencies of seal whiskers and the frequencies of underwater disturbances but also by the added mass and specific flow conditions, such as the angle of attack of the seal whisker to the incoming flow.^[^ ^5^ ^]^ Furthermore, it is worthwhile to investigate the quantitative relationship between the natural frequency and whisker morphology (further discussed in *Part C* of the Supporting Text, Supporting Information).

### 2.7 Modeling of 3D Seal Whiskers

**Figure 7:**
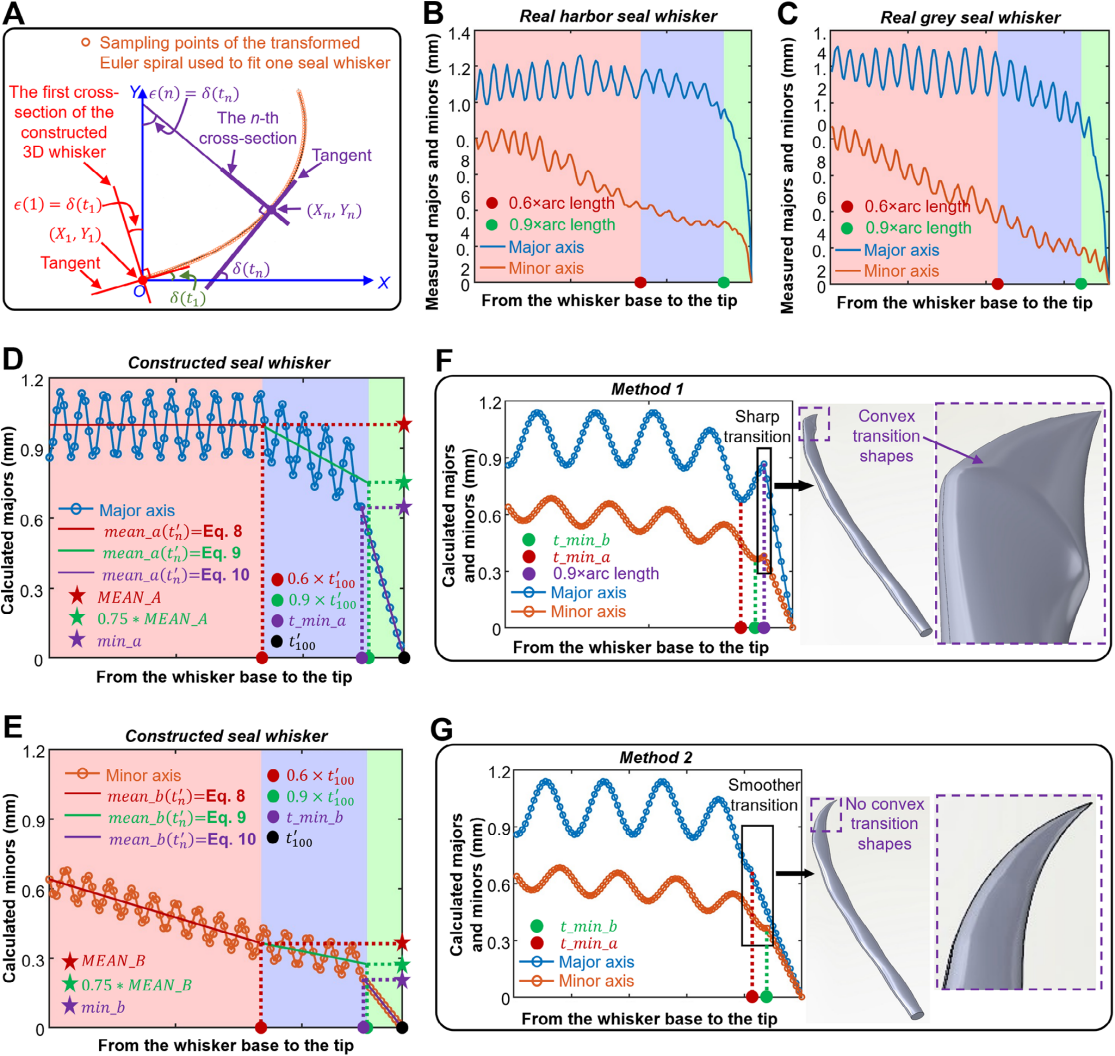
Calculation of the orientation angle and major and minor axes of cross‐sections from the whisker base to the tip. A) Definition of the orientation angle (*ϵ*(*n*)) of the *n*‐th cross‐section. B) Major and C) minor axes of one real seal whisker in three whisker segments: 1) 0–0.6× arclength of the whisker; 2) 0.6–0.9× arclength; and 3) 0.9–1× arclength. Adapted from.^[^ ^21^ ^]^ Copyright 2023 Wiley. Calculated D) major and E) minor axes of the constructed CAD model of the harbor seal whisker located at Column *G_L_*, Row *3*. F,G) Two different methods for calculating the major and minor axes of the harbor seal whisker located at Column *B_R_*, Row *4*, and the resulting seal whisker structures with and without convex transition shapes. The constructed seal whiskers based on Methods 1 and 2 are provided in Dataset S4 (Supporting Information).

**Figure 8:**
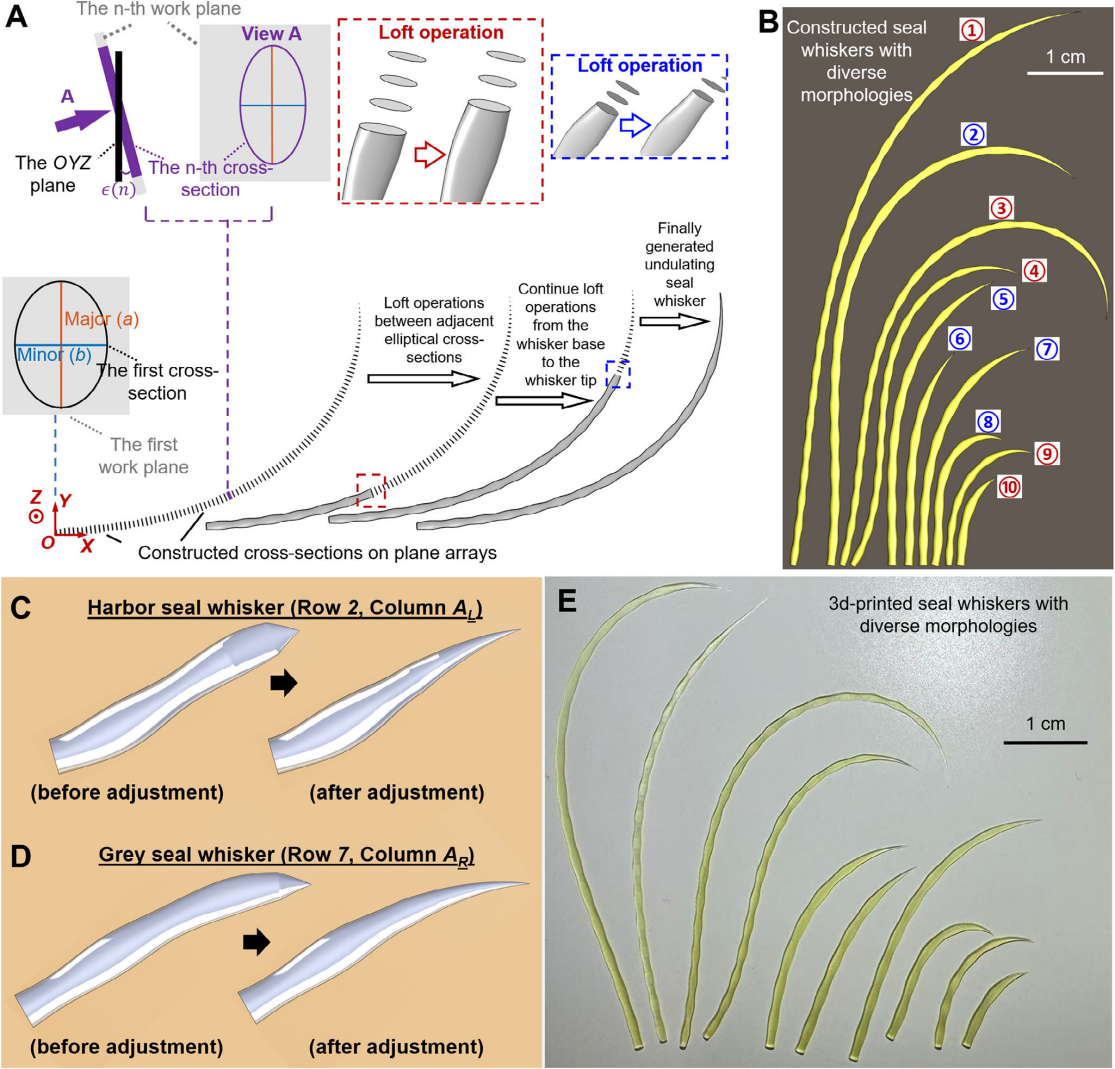
Construction of 3D wavy seal whiskers based on transformed Euler‐spiral‐fitted curves. A) Constructing a 3D seal whisker using lofting operations between adjacent cross‐sections along the whisker axis. B) CAD models of constructed seal whiskers with diverse length, thickness, and curvature values. In Movie S1 (Supporting Information), we rotate the 3D CAD models to show more details. The red serial number represents harbor seal whiskers, and their rows and columns are: ①: (Column *K_L_*, Row *2*); ③: (Column *I_L_*, Row *2*); ④: (Column *H_L_*, Row *1*); ⑨: (Column *C_L_*, Row *1*); and ⑩: (Column *A_R_*, Row *5*). The blue serial number represents grey seal whiskers, and their rows and columns are: ②: (Column *D_L_*, Row *1*); ⑤: (Column *C_L_*, Row *1*); ⑥: (Column *C_R_*, Row *1*); ⑦: (Column *E_R_*, Row *1*); and ⑧: (Column *A_L_*, Row *2*). All CAD models of 84 harbor seal whiskers and 57 grey seal whiskers are provided in Dataset S4 (Supporting Information). C,D) Short seal whiskers, including harbor seal whisker located at (Row *2*, Column *A_L_*) and grey seal whisker located at (Row *7*, Column *A_R_*), constructed using two different methods of segmenting the full‐length whisker. The “before adjustment” means the seal whisker was segmented with the first segment from 0–0.6× the whisker length, the second segment from 0.6–0.9× the whisker length, and the third segment from 0.9–1× the whisker length. The “after adjustment” means the three segments were adjusted, and the seal whisker has the first segment from 0–0.4× the whisker length, the second segment from 0.4–0.7× the whisker length, and the third segment from 0.7–1× the whisker length. E) Seal whisker prototypes fabricated using the BMF 3D printer.

As mentioned earlier, seal whiskers could be fitted using the transformed Euler spirals (Figure 2H). Using one transformed Euler spiral, we investigated the construction of a 3D seal whisker (*Part D* of the Experimental Section). Here, the transformed Euler spiral became the centerline of the constructed 3D seal whisker. First, we constructed all elliptical cross‐sections of the 3D seal whisker along the transformed Euler spiral (*Part D* of the Experimental Section). The morphological parameters—including the centroid coordinates, orientation angle (**Figure** **7**A), and the major and minor axes (Figure 7B–G) of cross‐sections—which are required for constructing 3D seal whiskers, were calculated (based on Equations (7)–(14)) from the start to the end points of the transformed Euler spirals. Thereafter, lofting operations were performed between adjacent cross‐sections from the whisker base to the tip (**Figure** **8**A and *Part D* of the Experimental Section) to construct CAD models (Figure 8B) of 3D seal whiskers.

Our previous measurements (Figure 7B,C)^[^ ^21^ ^]^ of the major and minor axes of cross‐sections of real harbor and grey seal whiskers revealed that the major axes oscillated around approximately constant values within approximately 0–0.6× the whisker length of the seal whiskers. This characteristic of the major axis has also been observed by Murphy et al.^[^ ^5^ ^]^ Moreover, the major axes of real seal whiskers continually decreased to zero (accompanied by a sine‐like oscillation) but had different reduction rates within approximately 0.6–0.9× the whisker length and 0.9–1× the whisker length of the seal whiskers. The minor axes of the real seal whiskers continually decreased to zero (accompanied by a sine‐like oscillation) but had different reduction rates within approximately 0–0.6× the whisker length, 0.6–0.9× the whisker length, and 0.9 to 1× the whisker length. Consequently, we divided the 3D seal whisker (to be constructed) into three whisker segments by whisker length (calculated using Equation (8)), after which we calculated the major and minor axes (calculated using Equations (9)–(14)) in three whisker segments, respectively (*Part D* of the Experimental Section). The equations (Equations (9)–(14)) used to calculate the major and minor axes in each whisker segment satisfied their variation laws (Figure 7B,C; described above) along the whisker length. Additionally, Equation (9), which was used to calculate the major and minor axes in the first seal whisker segment (approximately 0–0.6× the whisker length), was proposed in our previous work^[^ ^18^ ^]^ based on the fitting of measurements of the major and minor axes from the base to the tip of real seal whiskers (five harbor and five grey seal whiskers). Moreover, when constructing cross‐sections (perpendicular to the whisker axis) along the whisker length, we considered their orientation angles (*Part D* of the Experimental Section). The orientation angles were calculated using Equation (7), which has a simple mathematical form derived from the Euler spiral theory. Calculating the orientation angles of the cross‐sections was one of the motivations for investigating the Euler spiral in this study. Although we only used a finite number (i.e., 100) of cross‐sections located at finite (discrete) points (i.e., 100) on the whisker axis to construct the whisker, theoretically, we can calculate the orientation angles of an infinite number of cross‐sections located at infinite (continuous) points on the whisker axis, based on Equation (7).

Liu et al.^[^ ^41^ ^]^ constructed full‐length (from the whisker base to the tip) 3D seal whiskers using the reported data,^[^ ^5^, ^32^, ^42^, ^43^ ^]^ the shape of the whisker axis (reflecting the curvature characteristic of the seal whisker) was defined according to a cubic function (proposed by Graff et al.^[^ ^32^ ^]^) describing the relationship between the deflection and arc length. Additionally, the mean values (measured by Rinehart et al.^[^ ^42^ ^]^) of the major and minor axes at the crest and trough positions of the undulating surface of the seal whisker, and the mean wavelength of the undulation, were used to construct a simplified straight elliptical cylinder segment. Moreover, the variation in the cross‐sectional area from the whisker base to the tip, measured by Murphy et al.,^[^ ^5^ ^]^ was used to define the tapering characteristics of the seal whisker. By contrast, Graff et al.^[^ ^43^ ^]^ defined a tapered multiplier to scale down the major and minor axes of cross‐sections of seal whiskers from their maximum values at the whisker base to 25% of their maximum values at the whisker tip, accompanied by a cosine‐like oscillation of the major and minor axes. This operation equated the variations of the major and minor axes of cross‐sections of seal whiskers from the base to the tip in a continually decreasing process, accompanied by cosine‐like oscillations. Compared with^[^ ^41^ ^]^ and,^[^ ^43^ ^]^ the shape of the seal whisker axis was defined by the transformed Euler spiral that fitted the coordinates of the centerline of the realistic seal whisker. We used the transformed Euler spiral to calculate the orientation angle of the cross‐sections along the whisker length based on the equation (Equation (4) in the main text) derived from the Euler spiral theory. Additionally, the major and minor axes of all cross‐sections used to construct the 3D seal whisker were calculated from the whisker base to the tip based on Equations (9) and (10) (in the main text), which were proposed based on measurements of real seal whiskers in our previous study.^[^ ^18^ ^]^ The equations (Equations (9)–(14) in the main text) reflect the different variation laws described above of the major and minor axes in three segments along the whisker length, which were observed based on major and minor measurements of real seal whiskers.^[^ ^5^, ^18^ ^]^ In this study, we further realized the application of the equations (Equations (9) and (10) in the main text) we had previously proposed^[^ ^18^ ^]^ through the task of constructing 3D whiskers.

However, our method of constructing 3D full‐length seal whiskers with tapers, curvatures, and undulations has two limitations, as follows:

1. When calculating the major and minor axes of the constructed seal whiskers, we segmented the seal whiskers at 60% and 90% of their length; however, the 60% and 90% values were estimated based on our measurements (Figure 7B,C) of the major and minor axes of one real harbor seal whisker and one real grey seal whisker. The measurements based on more whiskers will provide us with more accurate varying trends of the major and minor axes of real seal whiskers from base to the tip, thus determining the segment ranges;
2. The fluctuations of major and minor axes are not obvious for some real seal whiskers with short lengths (<15 mm). However, based on our method, every constructed seal whisker exhibits fluctuations in the major and minor axes from the base to the tip. In addition, the different trends of the major and minor axes in the three whisker segments may not be obvious for some real short (<15 mm) seal whiskers. Moreover, for a short whisker, when using a segmented calculation method for the major and minor axes of the short whisker, its three whisker segments were all short. In this case, their major and minor axes decreased to zero in a very short segment (0.9–1× the whisker length) near the tip, which resulted in the sharp transition shapes (Figure 8C,D) near the tips of whiskers such as harbor seal whiskers located at (Row *2*, Column *A_L_*) and grey seal whiskers located at (Row *7*, Column *A_R_*). The convex transition shapes mentioned above can be solved by re‐segmenting the seal whisker to increase the length range of the third segment, for example, increasing it from 0.9–1× the whisker length to 0.7–1× the whisker length (which can be easily realized by just modifying the segment point in Code S3, Supporting Information) when calculating the major and minor axes used for constructing 3D seal whiskers. In this way, the major and minor axes in the third segment can decrease to zero in a longer segment length, smoothing the sharp transition shapes (Figure 8C,D). However, in Dataset S4 (Supporting Information), the third whisker segment of all harbor and grey seal whiskers is 0.9–1× the whisker length. We did not specially adjust the third whisker segment of short seal whiskers (<15 mm) to 0.7–1× the whisker length, maintaining consistency with seal whiskers of other lengths. In the future, we can determine the segmentation points accurately through measurements of full‐length short whiskers and then construct more accurate CAD models for short seal whiskers (<15 mm) using Codes S3 and S4 (Supporting Information).

Moreover, the equations (Equations (9) and (10) in the main text) were proposed based on measurements of five harbor seal whiskers with a length of 86.9 ± 9.43 mm and five grey seal whiskers with a length of 70 ± 3.39 mm.^[^ ^18^ ^]^ As discussed in *Part C* of the Results and Discussion section, the whisker thickness is highly correlated to the whisker length. Hence, the major and minor axes calculated based on Equations (9) and (10) (in the main text) are large for short whiskers. Based on the above, our method has limitations in constructing 3D CAD models of short seal whiskers (<15 mm).

The constructed seal whiskers in Dataset S4 (Supporting Information) can be scaled in CAD software and fabricated using 3d printers with different printing accuracies. In this work, we used the BMF 10‐µm Series Printer microArch S240 3D printer (with an optical resolution of 10 µm, a layer thickness of 20 µm, and a maximum print size of 100 (L) × 100 (W) × 75 (H) mm) to fabricate prototypes (Figure 8E) of ten constructed harbor and grey seal whiskers with diverse morphologies (length, thickness, and curvature). The prototypes (which were the same size as the CAD models) showed that the curvature, taper, and undulation characteristics of the seal whisker could be replicated, thereby validating the 3D printability of the morphological model. From a computational fluid dynamics standpoint, the provided CAD models (Dataset S4, Supporting Information, suitable for 3D printing) enable hydrodynamic simulations elucidating flow‐sensing mechanisms through constructed seal whiskers (including 84 harbor seal whiskers and 57 grey seal whiskers) as demonstrated in Figure 1E.^[^ ^10^, ^17^, ^18^ ^]^ Graff et al.^[^ ^32^ ^]^ comprehensively measured the spatial orientations (described by three Euler angles) of seal whiskers on the muzzle of the harbor seal. The three Euler angles were formulated as equations of whisker locations described by spherical coordinates. These equations provide valuable reference information regarding the spatial distribution of seal whiskers, which has contributed enormously to the research community, allowing researchers to distribute CAD models of 3D seal whiskers on a seal muzzle model to create a morphological model^[^ ^32^, ^41^, ^44^ ^]^ that can be used to investigate seal whiskers in an array of diverse research topics (Figure 1E–G), such as fluid dynamics.^[^ ^41^, ^44^ ^]^ From an experimental fluid dynamics standpoint, the 3D‐printed prototypes of seal whiskers facilitate experimental investigations of morphology‐dependent vibration responses (Figure 1F)^[^ ^19^, ^20^, ^21^ ^]^ providing mechanistic insights into the biological functions underlying wavy whisker structures (biological standpoint). From a sensor development standpoint, 3D‐printed prototypes of seal whiskers with diverse natural frequencies offer sensor development potential^[^ ^13^, ^20^, ^21^, ^22^, ^23^, ^24^, ^25^, ^26^, ^27^ ^]^ for specific sensory ranges (Figure 1G), advancing functional understanding of whisker‐based sensory systems.

## 3 Conclusion

In this study, we measured the morphologies and natural frequencies of two seal whisker species. We arranged the measurements on whisker location maps representing the actual distribution of seal whiskers on the seal muzzle. We then comprehensively analyzed the varying trends of the measurements from rostral to caudal. The databases of measurements (morphologies and natural frequencies) can guide the length and curvature design of seal‐whisker structures for whisker‐inspired sensors^[^ ^13^, ^20^, ^21^, ^22^, ^23^, ^24^, ^25^, ^26^, ^27^ ^]^ enabling the customization of specific sensing frequency ranges. Furthermore, our measurements of the natural frequencies revealed that whiskers at different locations on the seal muzzle responded to varying fishtail‐eddy frequencies.

To facilitate future studies on seal whiskers, we created 3D seal whiskers, including 84 harbor seal whiskers and 57 grey seal whiskers (Dataset S4, Supporting Information), based on major and minor axes (calculated by equations proposed by us^[^ ^18^ ^]^) of the ellipse‐fitted whisker cross‐sections, Euler‐spiral‐fitted whisker axes, and Euler‐spiral‐calculated orientation angles of whisker cross‐sections along the axes. The latter two morphological parameters and whisker curvatures were determined using the method proposed in this study, which enabled us to realize simultaneous fitting and mapping of seal whiskers based on Euler spirals. This method is an improvement over the one described in the literature,^[^ ^33^, ^34^ ^]^ which required two separate mathematical modeling steps for fitting and mapping. Additionally, Luo and Hartman's comprehensive examinations of mapping 114 harbor seal whiskers onto the standard Euler spiral^[^ ^36^ ^]^ showed that whiskers mapped on a standard Euler spiral lost all information regarding the whisker row and column identity. In this study, we showed that the fitting and mapping of seal whiskers based on the Euler spiral had significant value in calculating the average curvature of full‐length seal whiskers and cross‐sectional orientation angles used for constructing 3D seal whisker models. Moreover, the proposed method for constructing a full‐length 3D whisker with undulation, curvature, and taper characteristics may serve as a reference method for other whisker species.

In the future, we will design 3D‐printed whisker arrays attached to sensor arrays (Figure 1G) that can be mounted on underwater robots to assist them in tracking neighboring robots^[^ ^45^ ^]^ by detecting hydrodynamic stimuli (such as the shedding frequencies encoded in the vortex wakes of neighboring robots).^[^ ^20^ ^]^ The established database of 3D seal whiskers with undulations, curvatures, and taper characteristics can support flow sensor development with biomimetic whisker structures (Figure 1G).

## 4 Experimental Section

### Length and Thickness Measurements of Seal Whiskers

In this study, the whisker length was calculated from the base embedded in the follicle sinus complexes (FSCs) to the whisker tip.^[^ ^46^ ^]^ The whisker length was measured using image recognition technology (Figure 2A). The process was as follows: Each seal whisker was located on a sheet of green A4 paper, and its actual size (coordinate values) was calculated based on a scale bar on the paper (Figure 2A). A Canon EOS 4000D camera was positioned with its lens perpendicular to the paper to capture the seal whisker (Figure 2A). The captured images were input into ImageJ software to identify each whisker as a greyscale image. Next, the coordinates of the sampling points on the whisker shaft (each identified greyscale image) were collected using the GetData Graph Digitizer software. All the identified seal whisker coordinates are available in Dataset S2 (Supporting Information). Finally, the whisker length was measured by calculating the distance between adjacent sampling points using point coordinates and then summing all the calculated point‐to‐point distances. The whisker thickness was obtained by averaging three measurements of the minor axis of the seal whisker at three locations ($\frac{1}{4}$, $\frac{1}{2}$, and $\frac{3}{4}$ of the whisker length away from the whisker base; Figure 2A) using a digital caliper (Kunzer 7EMS01, accuracy 0.01 mm).

### Fitting Seal Whiskers Using Euler Spirals

The following parameterized form describes a standard Euler spiral: Equation (1),^[^ ^34^ ^]^ where |*t*| denotes the arc length from one point (*x*(*t*),  *y*(*t*)) on the standard Euler spiral to the origin, and $\left(\begin{array}{c} x(t)\\ y(t) \end{array}\right)$ = $\;-(\begin{array}{c} x(-t)\\ y(-t) \end{array})$.

A positive *t*‐value corresponded to the right half of the Euler spiral (Figure 2D). A transformed Euler spiral could be obtained by scaling the standard Euler spiral (Equation (1)) by a factor *K*, rotating it by angle ψ, and moving the origin to point *P*_0 _(*x*_0_, *y*_0_) (Figure 2D). A transformed Euler spiral could be expressed using Equation (2).^[^ ^28^ ^]^ The transformed Euler spiral was used to fit one seal whisker. As discussed in *Part A* of the Experimental Section, coordinates of the centerline of each whisker were collected (Dataset S2, Supporting Information) based on the identified greyscale image of the whisker. Whiskers of different lengths have varying numbers of coordinates. To facilitate the batch fittings for all whiskers, the image identification technology‐collected coordinates were first uniformly resampled along the whisker length for each whisker. The total number of resampling points of one seal whisker was 100. Then, the coordinates of the first resampling point were subtracted from the coordinates of all 100 resampling points (conducted using Code S1, Supporting Information) such that the starting point of the seal whisker was located at the origin (Figure 2D). Finally, the resampling points of the image identified‐whisker could be expressed by the coordinate set $P_{w}=\left\{\left(x_{w_{1}},\;y_{w_{1}}\right),\;\left(x_{w_{2}},\;y_{w_{2}}\right),\;\text{…}\left(x_{w_{n}},\;y_{w_{n}}\right)\right\}$, where *n* = 100.

The coordinates of the points on the transformed Euler used to fit the seal whisker were defined as $P_{w}^{\prime }=\;\left\{\left(x_{w_{1}}^{\prime },\;y_{w_{1}}^{\prime }\right),\;\left(x_{w_{2}}^{\prime },\;y_{w_{2}}^{\prime }\right),\;\text{…}\left(x_{w_{n}}^{\prime },\;y_{w_{n}}^{\prime }\right)\right\}$. Correspondingly, there were 100 points on the transformed Euler that fitted one seal whisker. The point $\left(x_{w_{n}}^{\prime },\;y_{w_{n}}^{\prime }\right)$ on the transformed Euler spiral corresponded to the point (*x*(*t_n_*), *y*(*t_n_*)) on the standard Euler spiral (Figure 2D). The arc length from point *P*_0 _(*x*_0_, *y*_0_) to point $\left(x_{w_{n}}^{\prime },\;y_{w_{n}}^{\prime }\right)$ on the transformed Euler spiral equals *K*|*t_n_* |,^[^ ^28^ ^]^ where *t_n_* denoted the arc length from the origin of the standard Euler spiral to the point (*x*(*t_n_*), *y*(*t_n_*)) on the standard Euler spiral (Figure 2D). Positive *t_n_* had a positive curvature, and the spiral rotated clockwise as *t_n_* increases; negative *t_n_* had a negative curvature, and the spiral rotates clockwise. The curvature (τ(*t_n_*)) at the point $\left(x_{w_{n}}^{\prime },\;y_{w_{n}}^{\prime }\right)$ of one transformed Euler spiral could be expressed using Equation (3).^[^ ^28^ ^]^ Additionally, the angle (δ(*t_n_*), Figure 2D) between the *ox* axis and tangent ([*cos*(δ(*t_n_*)), *sin*(δ(*t_n_*))]) at the point $\left(x_{w_{n}}^{\prime },\;y_{w_{n}}^{\prime }\right)$ could be expressed using Equation (4).^[^ ^28^ ^]^

(1) $$\left(\begin{array}{c} x\left(t\right)\\ y\left(t\right) \end{array}\right)=\left(\begin{array}{c} C\left(t\right)\\ S\left(t\right) \end{array}\right)=\left(\begin{array}{c} \int_{0}^{t}\cos\left(\frac{\pi \;\times \;u^{2}}{2}\right)du\\ \int_{0}^{t}\sin\left(\frac{\pi \;\times \;u^{2}}{2}\right)du \end{array}\right)\;$$

(2) $$\left(\begin{array}{c} x_{w}\left(t\right)\\ y_{w}\left(t\right) \end{array}\right)=K\left(\begin{array}{cc} \cos\left(\psi \right) & -\sin\left(\psi \right)\\ \sin\left(\psi \right) & \cos\left(\psi \right) \end{array}\right)\left(\begin{array}{c} C\left(t\right)\\ S\left(t\right) \end{array}\right)+\left(\begin{array}{c} x_{0}\\ y_{0} \end{array}\right)$$

(3) $$\tau \left(t_{n}\right)=\frac{\pi t_{n}}{K}\;$$

(4) $$\delta \left(t_{n}\right)=\;\;\psi +\frac{\pi {t_{n}}^{2}}{2}$$

Using the nonlinear fitting method—that is, the *nlinfit* function in the Statistics and Machine Learning Toolbox of MATLAB (Code S1, Supporting Information)— the point set (*P_w_*) was fitted to transformed Euler spirals (Figure 2D). The *nlinfit* function outputs six parameters (*K*, ψ, *t_start_*, *t_end_*, *x*_0_, *y*_0_), where *t_start_* (that is, *t*_1_ in the following) and *t_end_* (that is, *t*_100_ in the following) denote the arc lengths calculated from the origin of the standard Euler spiral to the start and end points, respectively, of the transformed Euler‐spiral‐fitted seal whiskers’ corresponding points on the standard Euler spiral. Once *t*_1_ and *t*_100_ were identified, the corresponding segment of the seal whisker on a standard Euler spiral—that is, the mapping result of the seal whisker on the standard Euler spiral—could be determined using Equation (1).^[^ ^34^ ^]^

The authors used the root‐mean‐squared error (*RMSE_w_*, Equation (5)) between the point sets of the seal whisker $(\;P_{w}=\;\;\left\{\left(x_{w_{1}},\;y_{w_{1}}\right),\;\left(x_{w_{2}},\;y_{w_{2}}\right),\;\text{…}\left(x_{w_{n}},\;y_{w_{n}}\right)\right\}$) and the transformed Euler spiral (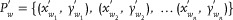) to evaluate the error of fitting one seal whisker using a transformed Euler spiral.

(5) $$RMSE_{w}=\sqrt{\frac{\sum_{n=1}^{100}\left({\left(x_{w_{n}}\;-\;x_{w_{n}}^{\prime }\right)}^{2}+{\left(y_{w_{n}\;}-\;y_{w_{n}}^{\prime }\right)}^{2}\right)}{100}}\;$$

As the curvature of the transformed Euler spiral‐fitted seal whisker varied linearly with the arc length (Equation (3)), the average value (denoted as $\bar{\tau }$, Equation (6)) of the curvatures was used at the start and end points to evaluate the curvature (Figure 4A) of the entire seal whisker.

(6) $$\bar{\tau }=\frac{\tau \left(t_{start}\right)+\tau \left(t_{end}\right)}{2}=\frac{\pi \left(t_{start}+t_{end}\right)}{2K}\;$$

### Natural‐Frequency Measurements of Seal Whiskers

The natural frequencies of the seal whiskers were measured using a laser Doppler vibrometer (LDV) system from Polytec Company.^[^ ^47^ ^]^ The LDV system could accurately measure the velocity and displacement of small, lightweight components without contact using a laser sensor. The velocity and displacement were characterized as recorded voltage signals. For the LDV‐aided measurements, the seal whisker was mounted in the air on a 3D‐printed holder of approximately 10 mm depth (Figure 2I) with soft encapsulation of the whisker base (elastic resin, Formlabs Company), which resembled the FSCs embedded in the seal muzzle.^[^ ^46^ ^]^ Additionally, the seal whisker was oriented with its minor axis parallel to the laser beam. The natural frequency was measured by manually plucking the seal whisker along its minor axis and recording the resulting voltage output of the laser sensor using the LDV system. The natural frequencies of the seal whiskers were measured by conducting a fast Fourier transform on the time‐series outputs of the laser sensor, and then noting the first dominant peak in the frequency domain. More details of the natural‐frequency measurements could be found in the previous works.^[^ ^20^, ^27^ ^]^ For a seal whisker with a length of less than 18 mm, only an extremely short whisker segment (less than 10 mm) protruded into the air (only the whisker tip in the air) after the whisker was mounted onto the 3D‐printed holder. Such short whisker segments did not vibrate (the tip was too rigid) or vibrate significantly (the tip was too soft) when plucked. Consequently, the natural frequencies of tiny seal whiskers could not be measured correctly, and each of them was not measured; whiskers that could vibrate significantly were only measured.

### Method of Constructing One 3D Full‐Length Seal Whisker

A coordinate system was defined as *O‐XYZ* (Figure 8A). The *OX* and *OY* axes were aligned with the plane of the paper and pointed to the right and the top, respectively. Additionally, the *OZ* axis was in the direction normal to the paper. The centerline of each constructed seal whisker was described using 100 points—that is, the 100 points of the corresponding transformed Euler spiral described in *Part B* of the Experimental Section. The coordinates of the first point were subtracted from the coordinates of all 100 points (conducted using Code S2, Supporting Information) so that the starting point of the constructed 3D seal whisker was located at the origin *O*. The obtained coordinates (Dataset S5, Supporting Information) were defined as (*X_n_*, *Y_n_*, *Z_n_*  =  0), where *n* = 100. The 100 points were uniformly distributed from the base (*X*_1_  =  0, *Y*_1_  =  0, *Z*_1_  =  0) to the tip (*X*_100_, *Y*_100_, *Z*_100_  =  0). A 3D seal whisker was constructed using 100 elliptical cross‐sections whose centers were located at the 100 points. Additionally, the origin *O* was the center of the first cross‐section of the constructed 3D seal whisker.

The 100 elliptical cross‐sections were constructed as follows: A transformed Euler‐spiral‐fitted seal whisker was located on the *OXY* plane. The transformed Euler‐spiral‐fitted seal whiskers were distributed along the positive directions of the *OX* and *OY* axes (Figure 8A). On the *OYZ* plane (the first work plane to draw the first cross‐section), the first elliptical cross‐section—that is, the whisker base was drawn. For the *n*th work plane on which the *n*th elliptical cross‐section was constructed, its center was located at the *n*th resampling point (*n*  = 1, 2, …, 100). The centers of the generated elliptical cross‐sections were located at the centers of the corresponding work planes. The *n* ^th^ work plane was normal to the *OXY* plane and to the whisker axis’ tangent vector at the *n*th resampling point (Figure 7A). It had an orientation angle of ε (*n*) (Dataset S5, Supporting Information) to the *OYZ* plane (Figure 7A). The parameter ε (*n*) of the *n*th cross‐section was equal to δ(*t_n_*). It was calculated using Equation (7) (realized using Code S2, Supporting Information), where the parameter *t_n_* denotes the arc length of the *n*th resampling point's corresponding point on a standard Euler spiral. The *t_n_* value was determined (realized using Code S1, Supporting Information) by fitting Equation (2) using the *nlinfit* function in the Statistics and Machine Learning Toolbox of MATLAB, as discussed in *Part B* of the Experimental Section.

(7) $$\varepsilon \;\left(n\right)=\delta \left(t_{n}\right)$$

Figure 7B,C shows the measured major (*a*) and minor (*b*) axes of the cross‐sections from the base to the tip of the real harbor and grey seal whiskers, respectively.^[^ ^21^ ^]^ The seal whisker was divided into three whisker segments along the arc length (Figure 7D,E). The variable $t_{n}^{\prime }$ in Equation (8) denotes the arc length (calculated from the first resampling point) of the *n*th resampling point on the transformed Euler‐spiral‐fitted seal whisker. It determined which whisker segment the *n*th resampling point was in. The value of $t_{100}^{\prime }$ denoted the full arc length of the seal whisker. The mean value of the major axis (*a*) was constant in the first whisker segment (0~0.6 × $t_{100}^{\prime }$). It decreased linearly by 25% in the second whisker segment (0.6 × $t_{100}^{\prime }$~0.9 × $t_{100}^{\prime }$) and finally dropped to zero in the third whisker segment (0.9 × $t_{100}^{\prime }$~1 × $t_{100}^{\prime }$). By contrast, the mean value of the minor axis (*b*) continually decreased with diverse rates (slopes) in the above three whisker segments.

(8) $$t_{n}^{\prime }=\left\{\begin{array}{c} 0\begin{array}{cc} , & n=1 \end{array}\\ \begin{array}{cc} t_{n\;-\;1}^{\prime }+\sqrt{{\left(X_{n}\;-\;X_{n\;-\;1}\right)}^{2}+{\left(Y_{n}\;-\;Y_{n\;-\;1}\right)}^{2}}, & n\;>\;1 \end{array} \end{array}\right.\;$$

In this study, the values of the major and minor axes were calculated in each of the three whisker segments separately. Specifically, the major (*a*(*n*)) and minor (*b*(*n*)) axes (Dataset S5, Supporting Information) of the *n*th cross‐section (realized using Code S3, Supporting Information) could be obtained using Equations (9)–(14). In the previous work,^[^ ^18^ ^]^ Equation (9) was proposed to calculate the major (*a*) and minor (*b*) axes in the first whisker segment (0–0.6 × $t_{100}^{\prime }$, Figure 7D,E), based on morphological measurements of elliptical cross‐sections of five real harbor seal whiskers and five real grey seal whiskers.^[^ ^18^ ^]^ The parameters, *V_k_* (*k *= 1, 2, …, 9), are presented in Table S1 (Supporting Information). The variables mean_a and mean_b were the mean values of the major and minor axes, respectively.

(9) $$\begin{array}{cc} & if\;0\;\leq \;t_{n}^{\prime }\;\leq \;0.6\;\times \;t_{100}^{\prime },\\ & \left\{\begin{array}{cc} a\left(t_{n}^{\prime }\right) & =V_{1}\;\times \;sin\left(V_{2}\;\times \;t_{n}^{\prime }+V_{3}\right)+V_{4}\\ b\left(t_{n}^{\prime }\right) & =V_{5}\;\times \;sin\left(V_{6}\;\times \;t_{n}^{\prime }+V_{7}\right)+V_{8}\;\times \;t_{n}^{\prime }+V_{9}\\ mean\_a\left(t_{n}^{\prime }\right) & =V_{4}\\ mean\_b\left(t_{n}^{\prime }\right) & =V_{8}\;\;\times \;0.6\;\times \;t_{n}^{\prime }+V_{9} \end{array}\right. \end{array}$$

By referring to the actual measurements (Figure 7B,C) on the seal whisker, the major and minor axes still had sine‐like fluctuations when their mean values decreased linearly in the second whisker segment (0.6 × $t_{100}^{\prime }$~0.9 × $t_{100}^{\prime }$). In the second whisker segment, the mean value of the major and minor axes decreased to two values equal to 75% of the mean_a and mean_b values corresponding to $t_{n}^{\prime }=0.6\;\times \;t_{100}^{\prime }$ (Equation (10)), which were defined as MEAN_A and MEAN_B, respectively. The mean_a and mean_b values plus sine functions with the same amplitude and phase as in Eq. 9 were used to describe the major axis $a(t_{n}^{\prime })$ and minor axis $b(t_{n}^{\prime })$ (Equation (10)) for the second whisker segment.

(10)

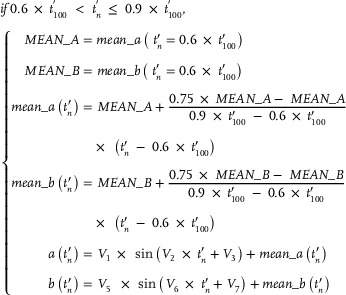




Regarding the major and minor axes in the third whisker segment (0.9 × $t_{100}^{\prime }$~1 × $t_{100}^{\prime }$), one method (Method 1) using a monotonically decreasing function (Equation (11)) was initially proposed to describe the major and minor axes of the third whisker segment. The major and minor axes decreased to zero from their values corresponding to $t_{n}^{\prime }=\;0.9\;\times \;t_{100}^{\prime }$. However, for some seal whiskers, the major and minor axes trends grew (as shown in Method 1) around the demarcation point (where $t_{n}^{\prime }=0.9\;\times \;t_{100}^{\prime }$) between the second and third segments. In such cases, significant sharp transitions could occur if the major and minor axes in the third whisker segment dropped directly to zero from the demarcation point (where $t_{n}^{\prime }=0.9\;\times \;t_{100}^{\prime }$). Such sharp transitions could cause convex transition shapes (around the demarcation point) on the surface of the constructed seal whisker (Figure 7F,G).

(11)

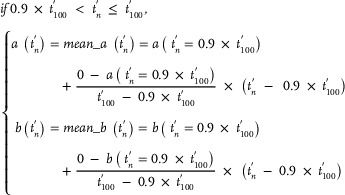




To solve this problem, the smallest values (*min_a* and *min_b* in Equation (12)) of the major and minor axes in the second whisker segment ($0.6\;\times \;t_{100}^{\prime }\;<\;t_{n}^{\prime }\;<\;0.9\;\times \;t_{100}^{\prime }$) were searched around the demarcation point between the second and third segments (Method 2). The major and minor axes in the third whisker segment then decreased from the *min_a* and *min_b* values (their corresponding arc lengths were t_min_a and t_min_b, respectively; Equation (12)) to zero (Equations (13) and (14)). Compared to Method 1, the treatment outlined in Method 2 achieved a smoother transition of the major and minor axes values from the second to the third whisker segments. In this case, the convex transition shapes on the surface of the constructed seal whisker were smoothed.

(12) $$\left\{\begin{array}{cc} \min\_a & =\min\left(a\left(0.6\;\times \;t_{100}^{\prime }\;<\;t_{n}^{\prime }\;<\;0.9\;\times t_{100}^{\prime }\right)\right)\\ \min\_b & =\min\left(b\left(0.6\;\times \;t_{100}^{\prime }\;<\;t_{n}^{\prime }\;<\;0.9\;\times \;t_{100}^{\prime }\right)\right)\\ t\_\min\_a\; & =t^{\prime }\;\left(a=\min\_a\right)\\ t\_\min\_b\; & =t^{\prime }\;\left(b=\min\_b\right) \end{array}\right.$$

(13) $$\begin{array}{cc} & if\;t\_min\_a\;<\;t_{n}^{\prime }\;<\;t_{100}^{\prime },\\ & a\left(t_{n}^{\prime }\right)=min\_a+\frac{0\;-\;min\_a}{t_{100\;}^{\prime }-\;t\_min\_a}\;\times \;\left(t_{n}^{\prime }\;-\;t\_min\_a\right) \end{array}$$

(14) $$\begin{array}{cc} & if\;t\_min\_b\;<\;t_{n}^{\prime }\;<\;t_{100}^{\prime },\\ & b\left(t_{n}^{\prime }\right)=min\_b+\frac{0\;-\;min\_b}{t_{100}^{\prime }\;-\;t\_min\_b}\;\times \;\left(t_{n}^{\prime }\;-\;t\_min\_b\right) \end{array}$$

After 100 cross‐sections were constructed, lofting operations (Figure 8B) between adjacent cross‐sections from the whisker base to the whisker tip (realized using Code S4, Supporting Information) were used to generate the 3D seal whiskers (Dataset S4, Supporting Information). Lofting operations^[^ ^48^ ^]^ were used in 3D modeling software applications (such as AutoCAD and SolidWorks) to generate smooth and continuous 3D shapes. In this study, lofting operations defined the desired undulating shape of the 3D seal whisker using 100 elliptical cross‐sections at the aforementioned 100 resampling points. COMSOL Multiphysics was integrated with MATLAB to realize the automatic procedures (using Code S4, Supporting Information) for constructing CAD models of 84 harbor and 57 grey seal whiskers. These procedures included the following: 1) Constructing all cross‐sections of one seal whisker from the whisker base to the whisker tip (see Code S4, Table S2, Supporting Information for the explanation of the corresponding codes); 2) using lofting operations between adjacent elliptical cross‐sections to generate one 3D seal whisker (see Code S4, Table S3, Supporting Information for the explanation of the corresponding codes). The detailed steps for integrating COMSOL Multiphysics with MATLAB to create a 3D wavy seal whisker were described in *Part D* of the Supporting Text (Supporting Information).

## Conflict of Interest

The authors declare no conflict of interest.

## Supporting information

Supporting Information

Supporting Information

Supporting Information

Supporting Information

## Data Availability

The data that support the findings of this study are available in the supplementary material of this article.;
