# Supplementary material for: Wonders of Harbor and Grey Seal Whiskers: Morphology, Natural Frequencies, and 3D Modeling
Source: Adv Sci (Weinh). 2025 Apr 30;12(23):2500724. doi: 10.1002/advs.202500724 (PMC12199420; doi:10.1002/advs.202500724)
Supplement: Supplementary file 1 — Supporting Information [file ADVS-12-2500724-s001.docx]

Supporting Information for

Wonders of Harbor and Grey Seal Whiskers: Morphology, Natural Frequencies, and 3D Modeling

Xingwen Zheng*, Amar M. Kamat, Ming Cao, Michael S. Triantafyllou, and Ajay Giri Prakash Kottapalli

* Email: xingwen.zheng@zju.edu.cn

**This PDF file includes:**

Supporting text

Figures S1–S4

Legends for Movie S1

Legends for Datasets S1–S5

Legends for Codes S1–S4

**Other supporting materials for this manuscript include the following:**

Datasets S1 to S5

Codes S1 to S4

Movie S1

Supporting Information Text

1. *Collections of seal whiskers and the definition of the whisker location map*

We collected whiskers from harbor (*Phoca vitulina*, female, age ~ two months) and grey seals (*Halichoerus grypus*, male, subadult) in Zeehondencentrum Pieterburen, The Netherlands. Whiskers on the left and right sides of the muzzles of both seals were collected. In this study, we examined whiskers of only one harbor seal and one grey seal. This can be improved in future studies, as we only examined two seals for two species rather than multiple seal groups, which could bias the research results. To denote the specific locations on the muzzle where each whisker was collected, the whiskers were successively plucked from the muzzle. Each time a whisker was plucked, a photograph showing its location on the seal muzzle was taken.

We defined one map (**Fig. 1H** in the main text) to describe the whisker location on the muzzle. The columns of the whisker location map are denoted by the symbols *A_R_*_,_ *B_R_*_,_ and *C_R_*, representing the progression from rostral to caudal (**Fig. 1H** in the main text). Correspondingly, the rows were designated as 1, 2, 3, …, indicating the transition from ventral to dorsal (**Fig. 1H** in the main text). We defined the locations of whiskers that adjoined the seal mouth as Row *1* and those that adjoined the seal nose and line connecting the center of the nose and mouth as Columns *A_R_* and *A_L_* (**Fig. 1I** in the main text), respectively. Other rows and columns adjoin the defined Row *1* and Columns *A_R_* and *A_L_*. We arranged the measured length, thickness, curvature, and natural frequency values of all seal whiskers in the defined whisker location map (**Fig. 1H** in the main text). In our previous investigations [1], we manually examined each photograph sequentially, cross-referencing the positions of whiskers in adjacent photographs to map the distribution of all seal whiskers on the muzzle. This approach relied on using the whisker position from the preceding photograph as a reference for the next. However, any error in identifying a whisker—particularly in areas (such as Row *1* of the left harbor seal muzzle) where whiskers are densely packed and hard to distinguish—would propagate to subsequent images, compounding inaccuracies. To address this, we adopted a new method in this study: comparing the whisker positions in each photograph directly to a single reference image (the first photograph in the sequence). This ensured a consistent reference point across all comparisons, significantly improving the accuracy of whisker localization.

It was found that some seal whiskers had been naturally lost (shedding), that they were broken or cracked (caused by plucking them), or that they were already used as components of whisker-sensors. These not-measured whiskers would not affect the qualitative varying trends of the measured natural frequency or whisker morphology, as only 1~2 whiskers were not measured per row and column (**Figs. 3** and **4** in the main text). In our previous investigations [1], we ignored the locations of these whiskers in each row of the whisker location map and made all whiskers in the same row close to each other without any interval. This helps to present the varying trend of measurements from rostral to caudal more clearly because whiskers in each row were continuously connected. In this study, to more accurately reflect the distribution of seal whiskers on the muzzle, we kept the locations of not-measured whiskers if they existed between measured whiskers in the defined whisker location map (**Fig. 1H** in the main text) and used empty ellipses to represent these whiskers in **Figs. 3** and **4** (in the main text).

1. *Analyses of the* $\bar{t}$ *and* $\Delta t$ *values*

When one seal whisker was mapped on the standard Euler spiral, the average ($\bar{t}=\frac{t_{start}+t_{end}}{2}$) of the $t_{start}$ and $t_{end}$ values reflected the location of the center of the seal whisker. The $\bar{t}$ values were located in the defined whisker location map (**Fig. S2**). The different $\bar{t}$ values in each column and each row (**Fig. S2**) indicated that the mapped whiskers were distributed at different positions on the standard Euler spiral at far and close distances from the origin. With a larger $\bar{t}$ value, the center of the mapped whisker is farther from the origin (**Fig. 2E** in the main text) of the standard Euler spiral than with a smaller $\bar{t}$ value. Moreover, there was no decreasing or increasing trend (as the variations of the whisker length and thickness) of the $\bar{t}$ values from rostral to caudal (**Fig. S3**), indicating that the locations (characterized by the $\bar{t}$ values) of seal whiskers mapped on the standard Euler spiral were not related to the seal whiskers’ length (**Fig. 3A** in the main text) and thickness (**Fig. 3B** in the main text), which increased from rostral to caudal. The irregularity of $\bar{t}$ values from rostral to caudal (**Fig. S3**) indicated that the location of the seal whisker mapped onto a standard Euler spiral could not reflect the row and column of one seal whisker on the muzzle. These observations match those reported for rat whiskers [2]. The difference ($\Delta t={t_{end}-t}_{start}$) between the $t_{start}$ and $t_{end}$ values corresponded to the distribution range of one seal whisker when mapped on the standard Euler spiral. In other words, the $\Delta t$ value represented the arc length of the seal whisker mapped on the standard Euler spiral. In addition, similar to the irregularity of $\bar{t}$ values, there was no decreasing or increasing trend of the $\Delta t$ values from rostral to caudal (**Fig. S3**).

1. *Quantitative relationship between the natural frequency and whisker morphology*

Based on the curve fitting methods, we formulated [1] the quantitative relationship between the natural frequency and seal-whisker morphology—including its length, thickness, and curvature. However, the aforementioned relationship has limitations because: 1) it does not consider the coupling relationships between the length, thickness, and curvature; and 2) it does not consider the fact that the natural frequency is related to not only the seal whisker morphology but also the material properties, such as the density and Young’s modulus [3]. The quantitative relationship between the natural frequency and seal-whisker morphology, as well as the material properties, can guide the length, thickness, and curvature design of seal-whisker structures for whisker-inspired sensors, ensuring the customization of specific sensing frequency ranges. Consequently, in the future, the quantitative relationship between the natural frequency and seal whisker-morphology, as well as the material properties, should be investigated from the perspective of physical mechanisms based on solid and fluid mechanical theories.

1. *The creation of one 3D wavy seal whisker*

In this article, we integrated COMSOL Multiphysics® with MATLAB® via the LiveLink™ for MATLAB® to realize the automatic procedures of the geometry creations in COMSOL Multiphysics®, including 1) Constructing all cross-sections of one seal whisker from the whisker base to the whisker tip (Code S4); 2) Using loft operations between adjacent elliptical cross-sections to generate one 3D seal whisker (Code S4). Methods and processes of 1) and 2) have been presented in the Method and Materials section of the main text. Tables S2 and S3 present every operation and corresponding codes in 1) and 2).

Taking 1), which used Code S4 to construct the CAD model of one entire seal whisker (Dataset S4), for example, steps that integrated COMSOL Multiphysics® with MATLAB® are as follows.

- Step 1: Install COMSOL Multiphysics® and Matlab softwares
- Step 2: Run COMSOL Multiphysics® Simulations with MATLAB®
- Step 3: Create file folders “harbor” and “transformedharbor” in :D drive.
- Step 4: Put files “orientation_angles_harbor.mat”, “centroid_x_coordinates_harbor.mat”, “centroid_y_coordinates_harbor.mat”, “major_axes_harbor.mat”, and “minor_axes_harbor.mat” in :D drive.
- Step 5: Run the MATLAB code. Seal whiskers will be generated automatically and saved as files.

1. *Additional description of Codes S1–S4 and Datasets S1–S5*

Legends for Codes S1–S4 and Datasets S1–S5 are presented on the last page of the supporting text. In Datasets S1-S5 and Codes S1-S4, we provide files titled "READ ME.txt" to describe the datasets and codes in detail.

The data files in Datasets S1-S5 have the following formats: 1) XLSX (can be opened in EXCEL), 2) CSV (can be opened in EXCEL), 3) PNG (can be opened in Microsoft Photos), 4) STEP (can be opened in CAD software, such as SOLIDWORKS), 5) SLT (can be opened in CAD software, such as SOLIDWORKS), and 6) MAT (can be opened in MATLAB).

The code files in Codes S1-S4 have .M format and can be opened in MATLAB. In Codes S1-S4, we include some data files in the same folders with the code files. These data files are necessary to run the code files, and they are all from Datasets S1-S5.

**SI References**

1. X. Zheng, Flow-sensing mechanisms and biomimetic potential of seal whiskers. Doctoral dissertation, University of Groningen (2022).
2. Y. Luo, M. J. Z. Hartmann, On the intrinsic curvature of animal whiskers. *PLOS One,* **18**, e0269210 (2023).
3. W. C. Young, R. Budynas, *Roark’s Formulas for Stress and Strain*. Seventh. New York, USA: McGraw-Hill Professional Publishing (2001).


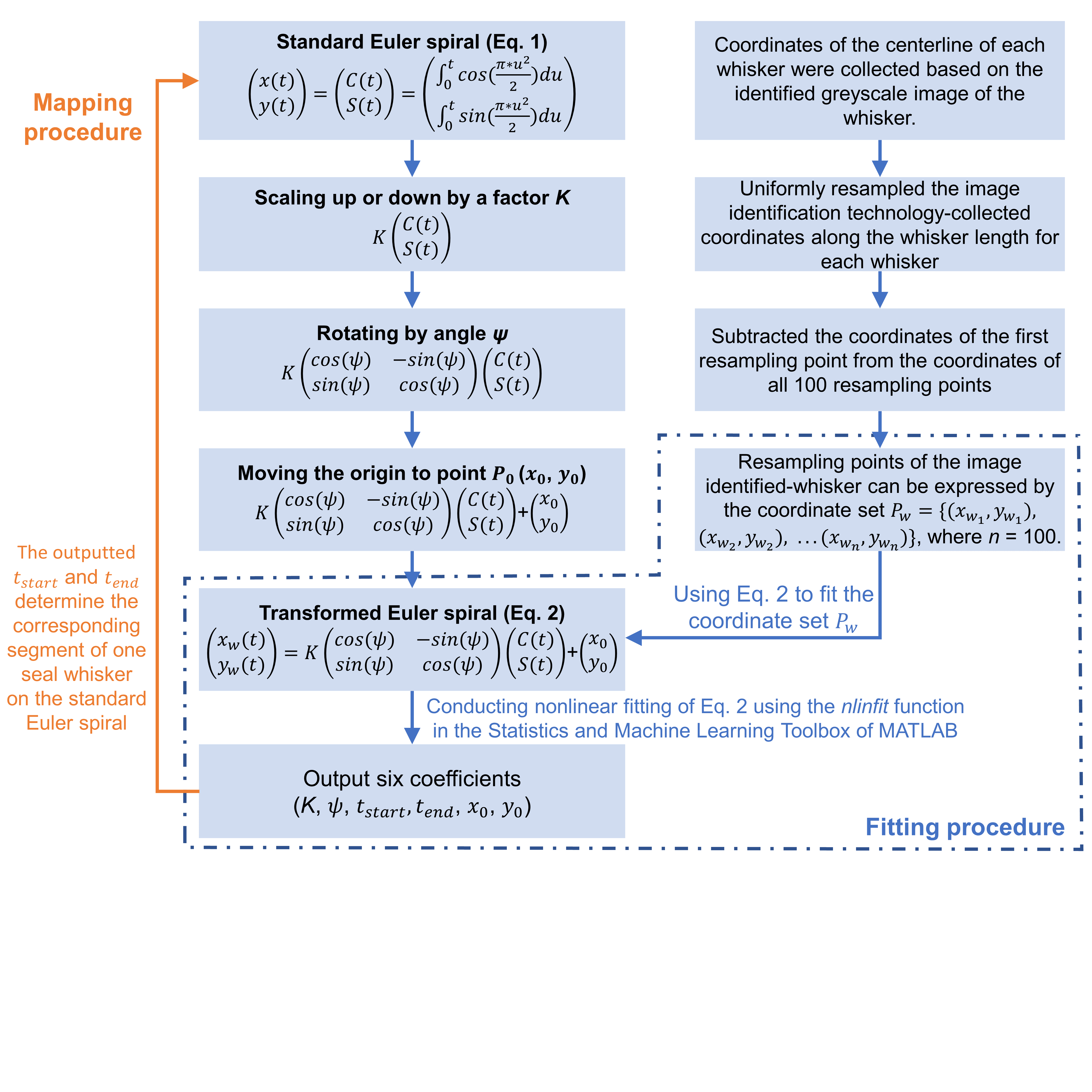


**Fig. S1 Flowchart of simultaneous fittings and mappings of seal whiskers based on Euler spirals**

**
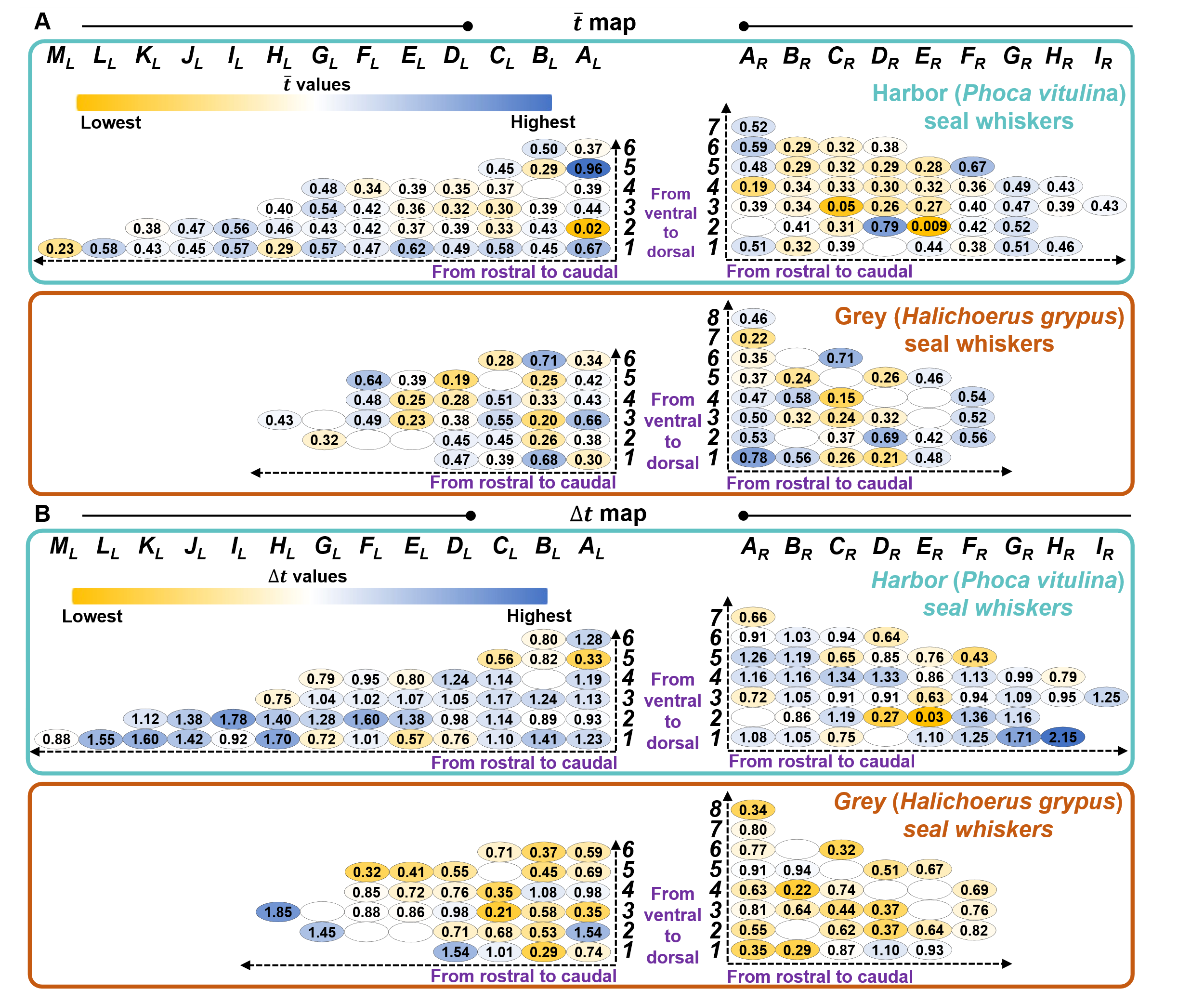
**

**Fig. S2. The average (**$\bar{\boldsymbol{t}}\boldsymbol{=}\frac{\boldsymbol{t}_{\boldsymbol{start}}\boldsymbol{+}\boldsymbol{t}_{\boldsymbol{end}}}{\boldsymbol{2}}$**) of the** $\boldsymbol{t}_{\boldsymbol{start}}$ **and** $\boldsymbol{t}_{\boldsymbol{end}}$ **values and the difference (**$\boldsymbol{\Delta t=}{\boldsymbol{t}_{\boldsymbol{end}}\boldsymbol{-t}}_{\boldsymbol{start}}$**) between the** $\boldsymbol{t}_{\boldsymbol{start}}$ **and** $\boldsymbol{t}_{\boldsymbol{end}}$ **values.** (**A**) The $\bar{t}$ value. (**B**) The $\Delta t$ value.

**
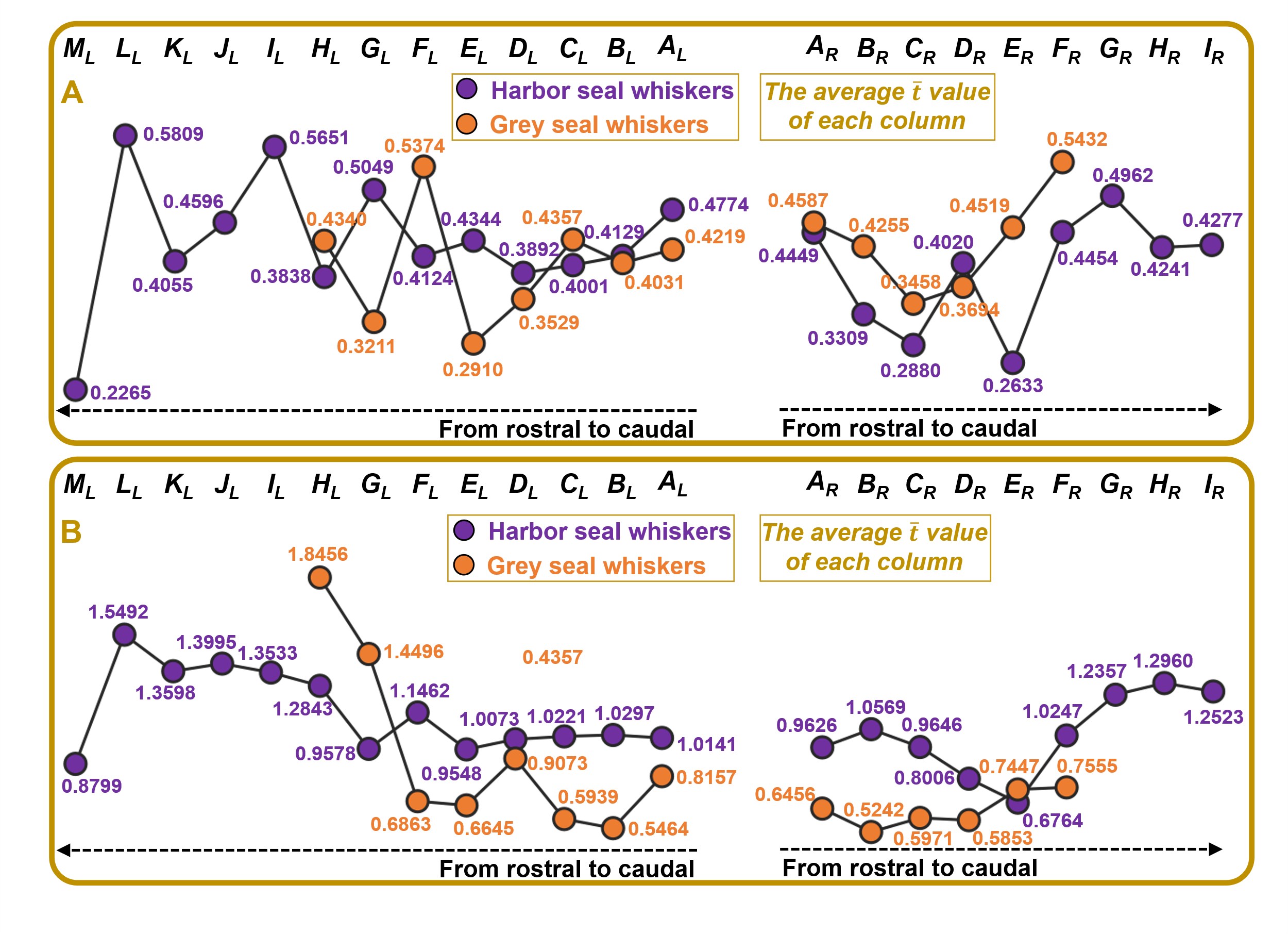
**

**Fig. S3.** **Varying trends of the** $\bar{\boldsymbol{t}}$ **and** $\boldsymbol{\Delta t}$ **values of seal whiskers** **from rostral to caudal on the seal muzzle.** (**A**) The varying trend of the $\bar{t}$ value. (**B**) The varying trend of the $\Delta t$ value.

**
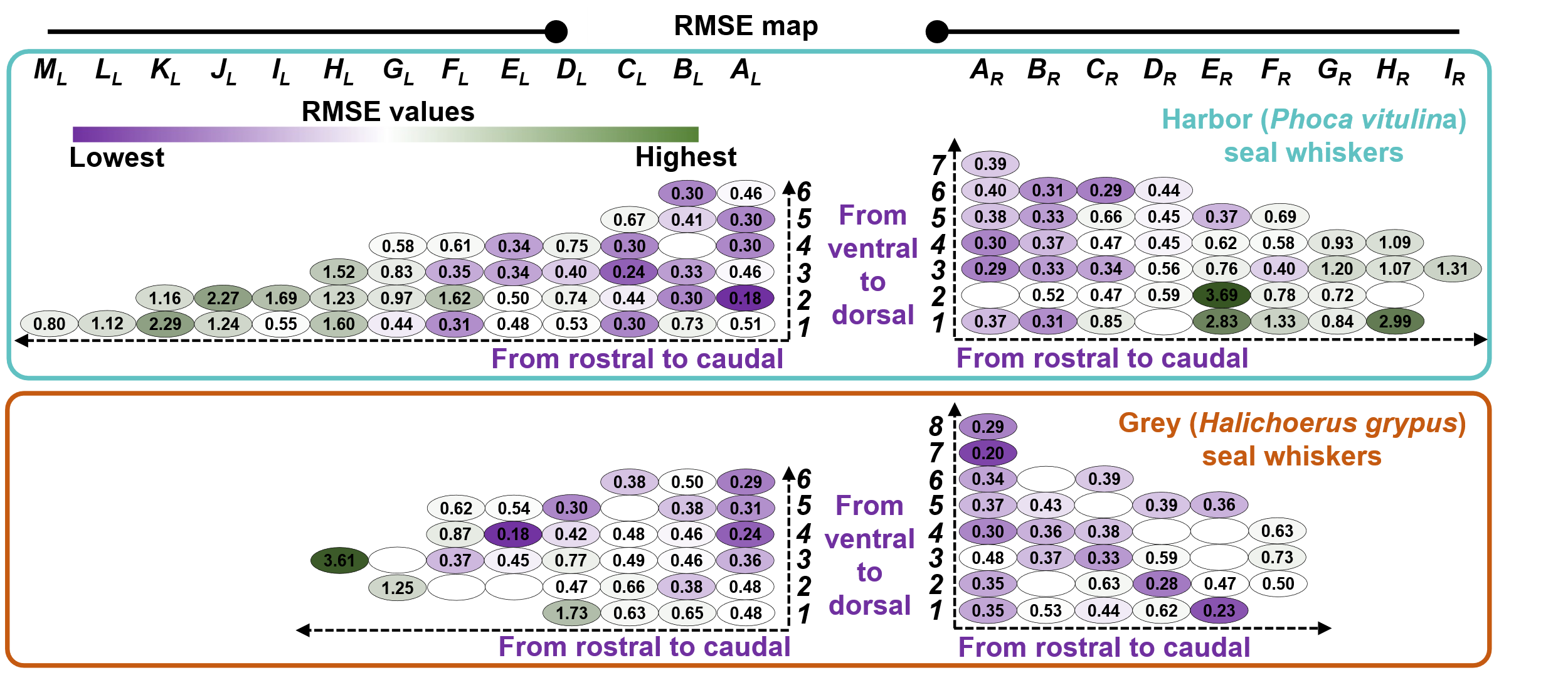
**

**Fig. S4. RMSE (mm) values between the point sets of the seal whisker and transformed Euler spiral used to fit the seal whisker**.

**Table S1. The geometrical framework parameters of harbor and grey seal whiskers**

| Item | Parameters | | | | | | | | |
| --- | --- | --- | --- | --- | --- | --- | --- | --- | --- |
| Harbor seal whisker | $V_{1}$ | $V_{2}$ | $V_{3}$ | $V_{4}$ | $V_{5}$ | $V_{6}$ | $V_{7}$ | $V_{8}$ | $V_{9}$ |
|  | 0.14 | 1.8 | 1.5$\pi$ | 1.0 | 0.067 | 1.8 | 1.0$\pi$ | –0.0082 | 0.64 |
| Grey seal whisker | $V_{1}$ | $V_{2}$ | $V_{3}$ | $V_{4}$ | $V_{5}$ | $V_{6}$ | $V_{7}$ | $V_{8}$ | $V_{9}$ |
|  | 0.13 | 1.4 | 1.0$\pi$ | 1.2 | 0.040 | 1.4 | 0.46$\pi$ | –0.0086 | 0.61 |

**Table S2. Explanation of Code S4 for the process of constructing all cross-sections of one seal whisker from the whisker base to the whisker tip.**

| **I. Constructing all cross-sections of one seal whisker from the whisker base to the whisker tip** | | | | |
| --- | --- | --- | --- | --- |
| 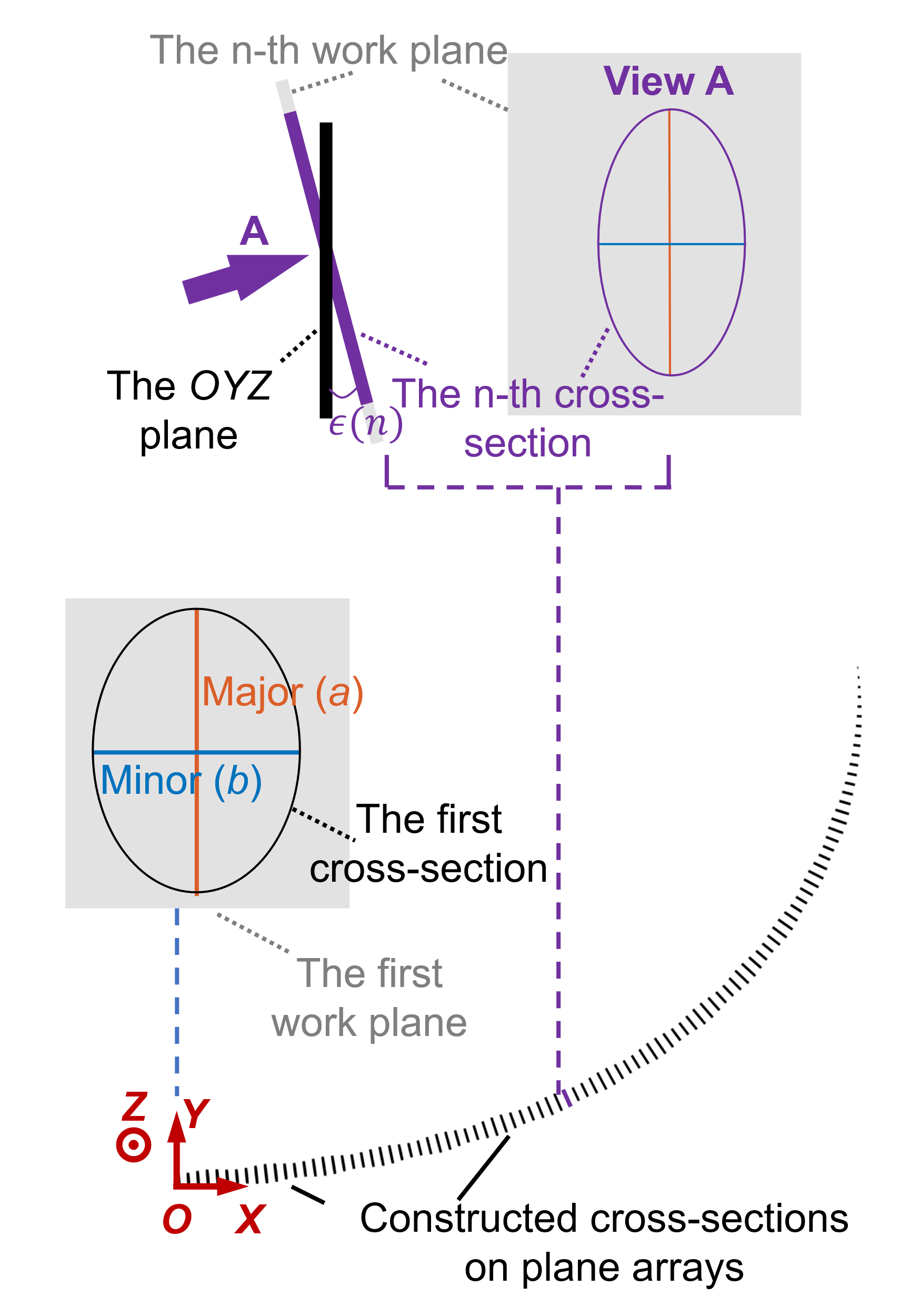 | **Parameters used to construct one seal whisker** | | **Calculating methods** | **Corresponding code files** |
|  | Majors of cross-sections *a* | | Equations 9-14 in the main text | generating_major_  and_minor_axes.m |
|  | Minors of cross-sections *b* | | Equations 9-14 in the main text | generating_major_  and_minor_axes.m |
|  | Orientation angles of cross-sections *ϵ* | | Equations 7 in the main text | data_generation.m |
|  | Centroid coordinates of cross-sections  (*X, Y, Z=0*) | | 100 resampling centroid coordinates (Dataset S5) from transformed Euler spirals of seal whiskers | data_generation.m |
| **Corresponding file** | | whiskergeneration_harbor.m and whiskergeneration_grey.m | | |
| **Operations** | | **Corresponding codes** | | |
| 1) The whisker base is pre-set to locate on the *OYZ* plane | | / | | |
| 2) Generating work planes (named ‘wp1’, ‘wp2’, ‘wp3’, ..., ‘wp100’) on which elliptical cross-sections will be generated later | | model.component('comp1').geom('geom1').create(strcat('wp', num2str(i)), WorkPlane'); | | |
| 3) The center of each generated work plane is set to locate at the coordinate point *(X, Y, Z =* 0*)* | | model.component('comp1').geom('geom1').feature(strcat('wp', num2str(i))).set('planetype', 'transformed');  model.component('comp1').geom('geom1').feature(strcat('wp', num2str(i))).set('transdispl', [x_center y_center 0]); | | |
| 4) Each work plane is set to have an orientation angle of *ϵ* | | model.component('comp1').geom('geom1').feature(strcat('wp', num2str(i))).set('transaxistype', 'z');  model.component('comp1').geom('geom1').feature(strcat('wp', num2str(i))).set('transrot', orientation_angle); | | |
| 5) Generating elliptical cross-sections with calculated majors *a* and minors *b* on corresponding work planes | | model.component('comp1').geom('geom1').feature(strcat('wp', num2str(i))).geom.feature(strcat('e_', num2str(i))).set('semiaxes', [semi_major semi_minor]); | | |
| 6) Centers of the generated elliptical cross-sections are located at the centers of corresponding work planes, which have global coordinates *(X, Y, Z =* 0*)* | | model.component('comp1').geom('geom1').feature(strcat('wp', num2str(i))).geom.feature(strcat('e_', num2str(i))).set('pos', [0 0]); | | |
| 7) The generated elliptical cross-sections are named ‘e1’, ‘e2’, ‘e3’, ..., ‘e100’. For one seal whisker, it will be constructed using 100 cross-sections | | model.component('comp1').geom('geom1').feature(strcat('wp', num2str(i))).geom.run(strcat('e_', num2str(i))); | | |

**Table S3. Explanation of Code S4 for the process of using loft operations between adjacent elliptical cross-sections to generate one 3D seal whisker.**

| **II. Using loft operations between adjacent elliptical cross-sections to generate one 3D seal whisker** | |
| --- | --- |
| 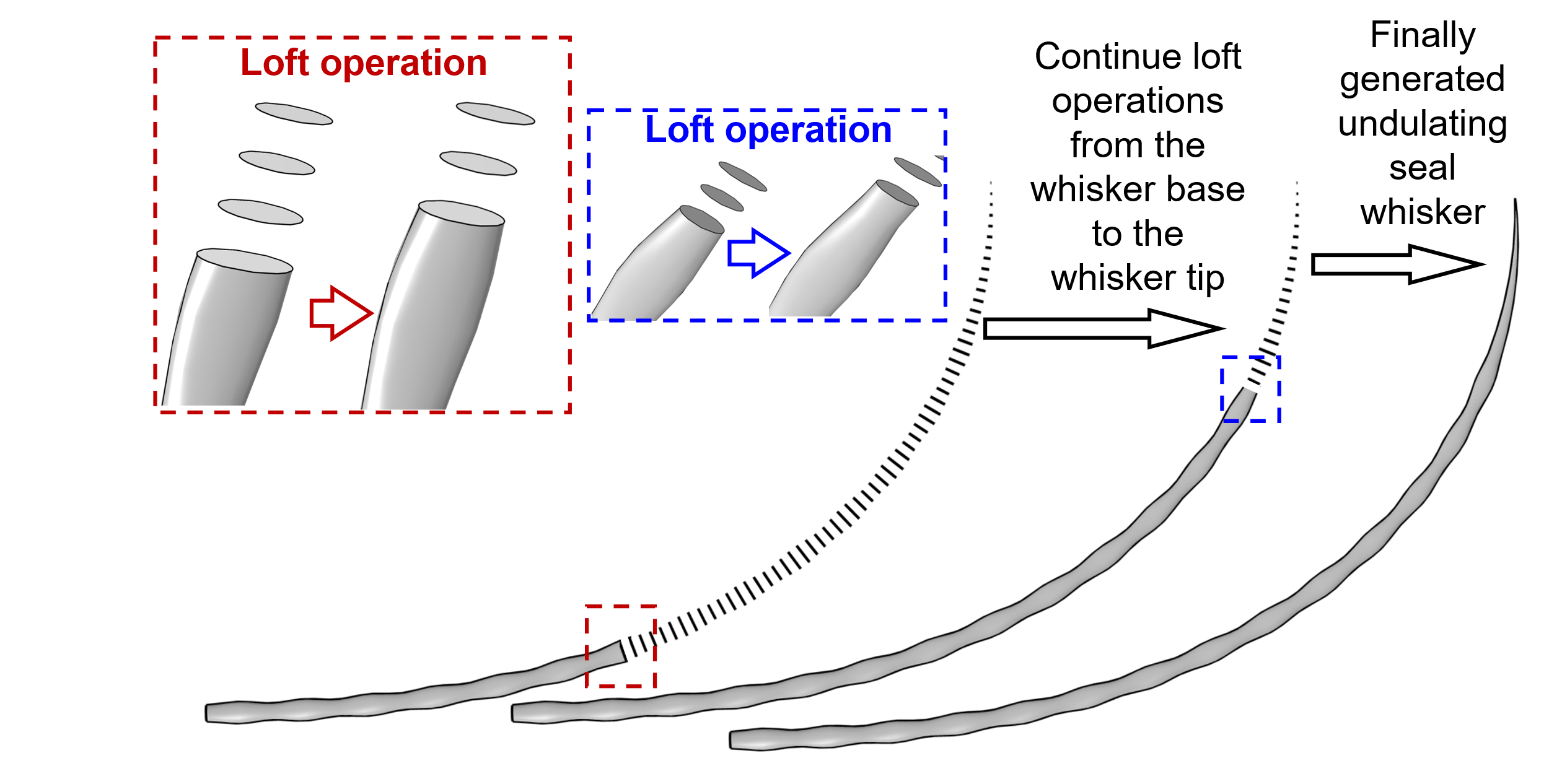 | |
| **Corresponding file** | whiskergeneration_harbor.m and whiskergeneration_grey.m |
| **Operations** | **Corresponding codes** |
| Loft operations between adjacent cross-sections from the whisker base to the whisker tip | model.component('comp1').geom('geom1').create('loft1', 'Loft'); model.component('comp1').geom('geom1').feature('loft1').selection('profile').set({'wp1' 'wp2' 'wp3' 'wp4' 'wp5' 'wp6' 'wp7' 'wp8' 'wp9' 'wp10' 'wp11' 'wp12' 'wp13' 'wp14' 'wp15' 'wp16' 'wp17' 'wp18' 'wp19' 'wp20' 'wp21' 'wp22' 'wp23' 'wp24' 'wp25' 'wp26' 'wp27' 'wp28' 'wp29' 'wp30' 'wp31' 'wp32' 'wp33' 'wp34' 'wp35' 'wp36' 'wp37' 'wp38' 'wp39' 'wp40' 'wp41' 'wp42' 'wp43' 'wp44' 'wp45' 'wp46' 'wp47' 'wp48' 'wp49' 'wp50' 'wp51' 'wp52' 'wp53' 'wp54' 'wp55' 'wp56' 'wp57' 'wp58' 'wp59' 'wp60' 'wp61' 'wp62' 'wp63' 'wp64' 'wp65' 'wp66' 'wp67' 'wp68' 'wp69' 'wp70' 'wp71' 'wp72' 'wp73' 'wp74' 'wp75' 'wp76' 'wp77' 'wp78' 'wp79' 'wp80' 'wp81' 'wp82' 'wp83' 'wp84' 'wp85' 'wp86' 'wp87' 'wp88' 'wp89' 'wp90' 'wp91' 'wp92' 'wp93' 'wp94' 'wp95' 'wp96' 'wp97' 'wp98' 'wp99' 'wp100'});  model.component('comp1').geom('geom1').run('loft1'); |

**Dataset S1 (separate file).** The raw data of length, thickness, curvature, natural frequency, RMSE, $\bar{t}$, and $\Delta t$ values of harbor and grey seal whiskers

**Dataset S2 (separate file).** The coordinates of the centerline of each whisker (collected using the GetData Graph Digitizer software)

**Dataset S3 (separate file).** All harbor and grey seal whiskers fitted by transformed Euler spirals and mapped on one standard Euler spiral

**Dataset S4 (separate file).** 3D CAD models (can be used for the 3d printing) of all constructed harbor and grey seal whiskers

**Dataset S5 (separate file).** The centroid coordinates, major axes, minor axes, orientation angles of all cross-sections for all harbor and grey seal whiskers

**Code S1 (separate file).** MATLAB code of fitting one curved seal whisker using one transformed Euler spiral and mapping one seal whisker on one standard Euler spiral

**Code S2 (separate file).** MATLAB code used to generate centroid coordinates, orientation angles, and arc lengths corresponding to all cross-sections for all harbor and grey seal whiskers

**Code S3 (separate file).** MATLAB code used to generate major and minor axes of all cross-sections of one entire seal whisker

**Code S4 (separate file).** MATLAB code used to construct the CAD model of one entire seal whisker

**Movie S1 (separate file).** Observing details of the 3D CAD models of the constructed seal whiskers.
